# A minimalistic cyclic ice-binding peptide from phage display

**DOI:** 10.1038/s41467-021-22883-w

**Published:** 2021-05-11

**Authors:** Corey A. Stevens, Fabienne Bachtiger, Xu-Dong Kong, Luciano A. Abriata, Gabriele C. Sosso, Matthew I. Gibson, Harm-Anton Klok

**Affiliations:** 1 Laboratoire des Polymères, Institut des Matériaux and Institut des Sciences et Ingénierie Chimiques, École Polytechnique Fédérale de Lausanne (EPFL), Lausanne, Switzerland; 2 Department of Chemistry and Centre for Scientific Computing, University of Warwick, Coventry, UK; 3 Laboratory of Therapeutic Proteins and Peptides, Institute of Chemical Sciences and Engineering, École Polytechnique Fédérale de Lausanne (EPFL), Lausanne, Switzerland; 4 Protein Production and Structure Core Facility and Laboratory for Biomolecular Modeling, École Polytechnique Fédérale de Lausanne (EPFL) and Swiss Institute of Bioinformatics, Lausanne, Switzerland; 5 Warwick Medical School, University of Warwick, Coventry, UK

**Keywords:** Peptides, Biopolymers

## Abstract

Developing molecules that emulate the properties of naturally occurring ice-binding proteins (IBPs) is a daunting challenge. Rather than relying on the (limited) existing structure–property relationships that have been established for IBPs, here we report the use of phage display for the identification of short peptide mimics of IBPs. To this end, an ice-affinity selection protocol is developed, which enables the selection of a cyclic ice-binding peptide containing just 14 amino acids. Mutational analysis identifies three residues, Asp8, Thr10 and Thr14, which are found to be essential for ice binding. Molecular dynamics simulations reveal that the side chain of Thr10 hydrophobically binds to ice revealing a potential mechanism. To demonstrate the biotechnological potential of this peptide, it is expressed as a fusion (‘Ice-Tag’) with mCherry and used to purify proteins directly from cell lysate.

## Abstract (web summary)

Developing molecules that emulate the properties of naturally occurring ice-binding proteins (IBPs) is a daunting challenge. Here, the authors demonstrate the use of phage display for the identification of short peptide mimics of IBPs, which resulted in the identification of a cyclic ice-binding peptide containing just 14 amino acids.

## Introduction

Ice-binding proteins (IBP) (also known as antifreeze proteins (AFP)) are produced in psychrophilic species as an evolutionary mechanism to enable survival in sub-zero climates^1^. These proteins achieve the remarkable molecular recognition feat of selectively binding ice in the presence of a large excess of water inducing the macroscopic properties of thermal hysteresis (non-equilibrium depression of freezing point), ice recrystallization inhibition (IRI) and dynamic ice shaping, all characteristic of macromolecule–ice interactions. There is large structural diversity between IBPs from the relatively large and rigid *Marinomonas primoryensis* AFP stabilized by extensive calcium binding to the smaller and flexible antifreeze glycoproteins (AFGPs)^1^.

There is a great interest to unravel and understand the interactions between IBPs and ice. This is not only of fundamental interest but can also help to identify design principles that facilitate the development of synthetic materials that emulate the ice-binding properties of IBPs. Analysis of the amino acid sequence and structure of IBPs has provided some insight into structure–function relationships^2^. For example, the AFGPs are based on a glycosylated tripeptide motif Ala-Ala-Thr, with naturally occurring variants ranging from 4–55 repeating tripeptide units (2.4–34 kDa). These naturally occurring IBPs have provided structural information that has guided the design of a variety of synthetic peptide-based analogues over the past years. The synthesis of IBP analogues, however, is often very challenging, in particular on a large scale, and activities are variable^3,4^.

In this work, we report a conceptually alternative approach to the development of synthetic IBP mimics. Rather than building upon existing structural know-how, this study explores the use of phage display, a biological, combinatorial discovery tool, for the identification of short peptide mimics of IBPs. Since the starting point of the phage display process is a randomized peptide library, this strategy vastly expands the diversity space that can be screened for potential ice-binding peptides. This may help to provide further structural insight into the key structural determinants that govern the properties of IBPs. Phage display, furthermore, generally leads to short peptides that are composed of naturally occurring amino acids, which may also enhance the synthetic accessibility of these compounds. Using phage display, a 14 amino acid, cyclic peptide ice recrystallization inhibitor is discovered. The solution structure of the peptide is characterized by NMR spectroscopy, and mutational analysis experiments reveal three residues, Asp8, Thr10 and Thr14, which are found to be essential for ice binding. Molecular dynamics (MD) simulations show that the peptide binds to ice adopting specific conformations through a combination of both hydrophobic interactions (Thr10 and Thr14) and hydrogen bonding (Asp8). Finally, to illustrate the potential use of short ice-binding peptides, the discovered sequence is used as a purification tag to extract overexpressed recombinant mCherry fluorescent protein from *Escherichia* *coli* cell lysate.

## Results

### Phage display and affinity selection

Phage display allows the discovery of high-affinity, short peptide ligands against a broad range of protein substrates^5–7^. Over the years, the scope of this technique has expanded and phage display has been successfully applied for the identification of short peptide ligands that can selectively bind to other targets including metals, semi-conductors, ceramics as well as a range of biological and synthetic polymers^8–15^. Ice, however, is an exceptionally challenging target substrate as it presents a dynamic interface. Ice is formed in water and is constantly growing/melting.

**Fig. 1:**
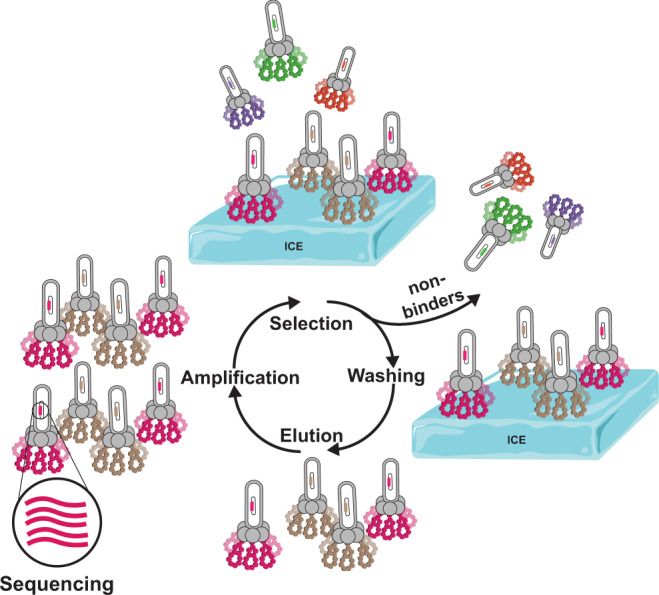
Phage display. Schematic illustration of the phage display process used for the identification of ice-binding peptides.

**Fig. 2:**
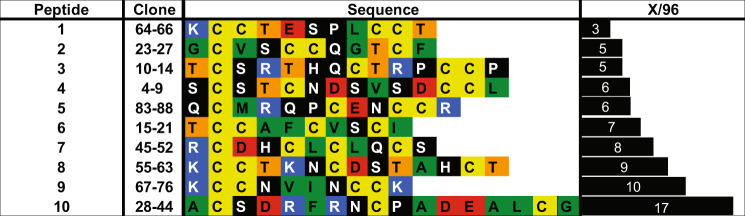
Most abundant phage clones identified by phage display. Listed are the amino acid sequences and frequencies of occurrence of the ten most abundant phage clones. Clone numbers correspond to those presented in Supplementary Table 1.

Figure 1 schematically illustrates the phage display process that was used to identify ice-binding peptides. For the experiments conducted in this study, phage libraries were constructed from a filamentous phage fd, which displays five copies of each peptide at the N terminus of the pIII coat protein at the end of the viral particle^16^. The phage library was designed to accommodate the formation of both mono- and bicyclic peptides^17^. The cyclic peptide library was generated by encoding four cysteines into each peptide while randomizing the remaining residues. Oxidation of all four cysteine residues can lead to the formation of two disulfide bridges and subsequently, a variety of bicyclic peptide structures. When only two cysteine residues are paired via a disulfide bridge, monocyclic peptides are obtained. Cyclic peptides have reduced conformational flexibility, which results in lower entropic penalties upon target binding, and increased diversity as compared to linear libraries of the same length^17,18^. To further increase diversity, a mixture of six different libraries varying in length from 9 to 14 amino acids was used. For the initial panning experiments, the mixture of phage libraries contained ~2 × 10^10^ unique peptides sequences^17^. For the affinity selection step, first an ice shell was formed inside a round bottom flask that was rotated in an ethylene glycol bath held at −0.5 °C. Then, a phage library solution was incubated with the ice for approximately 2 min to capture ice-binding phages. The incubation time was limited to just 2 min in order to prevent ice overgrowth and preserve phage function. After that, the remaining liquid, containing unbound phages, was decanted and the ice was washed with pre-cooled buffer solution. Next, the ice was melted, releasing bound phages that were subsequently used to infect *E. coli* to amplify phages available for iterative panning cycles. This strategy bears resemblance to the rotary ice-affinity purification method that has been used to isolate IBPs from organisms including bacteria, insects and plants^19–21^. By measuring the output phage titer after each round of selection, the enrichment of the phage library towards ice could be assessed. Repetition of the affinity selection cycle resulted in a gradual increase in the output phage titer. After five rounds, an ~15-fold increase in the output phage titer was determined (supplementary fig. 1). As there was no titre improvement in round 5, 96 phage clones were selected for sequencing from round 4. Figure 2 lists the amino acid sequences and the relative frequencies of occurrence of the 10 most abundant peptides that were identified. The full list of identified sequences is presented in Supplementary Table 1. **Peptide 10** in Fig. 2 is a result of the initial library design^22^. This sequence was used to generate the peptide library via random mutagenesis by PCR. As a consequence, a small amount of **peptide 10** remains in the library as a ‘contaminating sequence’. **Peptide 10** was not pursued further. **Peptides 1**–**9** listed in Fig. 2 were subsequently synthesized by solid-phase peptide synthesis and their respective HPLC and LC–MS data can be found in Supplementary Figs. 2–10.

**Fig. 3:**
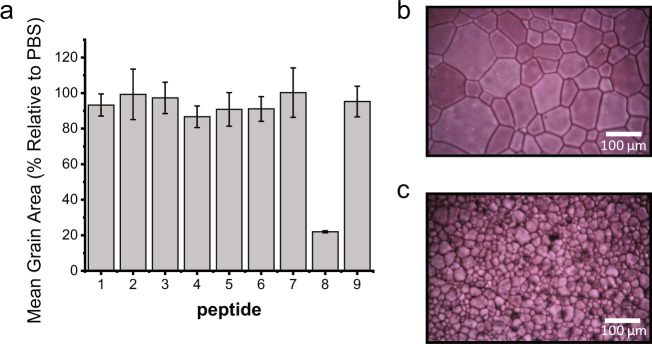
Ice recrystallization activities of the peptides listed in Fig. 2. **a** IRI activity of **peptides 1–9** tested at 1 mg/mL (values reported are averages from three individual experiments shown as mean data values ± SD. Error bars represent standard deviation within the repeats). **b**, **c** Representative cryomicrographs of ice crystals grown in the presence of 1 mg/mL of **peptide 3** (**b**) and **peptide 8** (**c**). Experiments were performed in triplicate.

As a first screen to assess their activity, **peptides 1**–**9** were evaluated for their IRI activity by the ‘splat’ assay^23,24^. This assay involves dropping a 10 μL aliquot of the peptide solution in phosphate-buffered saline (PBS) onto a glass coverslip, which is resting on an aluminium block cooled to −78 °C. This results in the instantaneous formation of a thin layer of ice, which is subsequently transferred to a cryostage and annealed at −8 °C for 30 min. After 30 min, the relative size of the ice crystals is evaluated and compared with those of a control buffer sample that does not contain a peptide. Figure 3a summarizes the IRI activities, expressed as mean grain area relative to the PBS control, for the nine identified peptides at a concentration of 1 mg/mL. Of the peptides, only **peptide 8** showed significant IRI activity. While all **peptides** **1–9** are based on sequences that have been identified via phage display, the absence of IRI activity for **peptides** **1–7** and **9** indicates that phage affinity does not necessarily translate into peptide affinity. One possible reason for this can be that binding of a phage, which presents five copies of the peptide, to ice can proceed via multivalent interactions, whereas **peptides 1–9** bind in a monovalent fashion. Figure 3b, c shows photographs of ice crystals formed in the presence of 1 mg/mL **peptide 3** and **peptide 8**, respectively, and illustrate the significant difference in ice grain size due to **peptide 8**.

**Fig. 4:**
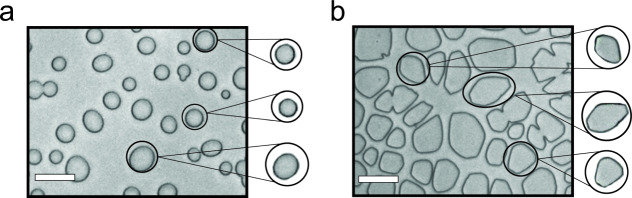
Ice shaping analysis of the peptides. The images show representative cryomicrographs of ice crystals grown in PBS containing 40% sucrose in the presence of **peptide 3** (**a**) and **peptide 8** (**b**) at 1 mg/mL (Scale bars correspond to 100 µm). Experiments were performed in triplicate.

To probe the interactions of **peptide 8** with ice, ice shaping experiments were performed. These experiments were carried out by sandwiching a droplet of a 1 mg/mL solution of **peptide 8** in PBS containing 40% (w/v) sucrose between two glass coverslips. These are subsequently transferred to a cold stage, which is rapidly cooled to −50 °C, and held for 2 min. Then, the ‘sandwich’ is warmed to −8 °C and annealed for 30 min. The presence of sucrose reduces nucleation and crystal growth, allowing for the monitoring of the growth and development of individual ice grains. Figure 4a shows a representative photograph of ice crystals grown in the presence of **peptide 3**. As can be seen, the individual ice crystals have a disc-like shape, which is typical of uninhibited ice growth. This observation indicates that **peptide 3** is unable to bind ice. The same disc-like shape present in Fig. 4a would be observed in the absence of peptide. Figure 4b depicts ice crystals that have been formed in a solution containing **peptide 8**. In this case, the ice crystals are faceted. Ice crystals become faceted when ice growth is inhibited in some way, like by bound IBPs or in this case **peptide 8**. This observation is important because ice-binding and IRI activity are not linearly related. There are many examples of compounds, like small AF(G)P mimics, that have high IRI activity but are unable to bind to ice^25^.

### Peptide solution structure

The structure of **peptide 8** was examined by solution NMR spectroscopy. NMR experiments were performed with 6 mg/mL **peptide 8** dissolved in 0.5× PBS at pH = 6.0 with 10% deuterium oxide (D_2_O). Such high concentration enables the acquisition of ^1^H, ^15^N and ^1^H, ^13^C heteronuclear single quantum coherence spectra at natural abundance, allowing for full characterization. Supplementary Figs. 11 and 12 present ^1^H**–**^15^N and ^1^H**–**^13^C correlation spectra that were recorded to assign the peptide backbone structure. Supplementary Table 2 summarizes the atom specific chemical shifts determined from these experiments. Nuclear Overhauser Effect spectroscopy experiments performed with 120 or 200 ms mixing times, at two fields (500 and 800 MHz ^1^H Larmor frequency) and at different temperatures, revealed very few NOEs. All NOE crosspeaks were found to arise from consecutive residues with no long-range H,H correlations (Supplementary fig. 13). Rotating Frame Nuclear Overhauser Effect spectra did not provide further peaks either. These observations are indicative of a rather flexible peptide structure. Since the C_β_ chemical shifts of the cysteine residues are sensitive to the redox state of the thiol side chain functional groups, ^13^C-NMR analysis allows to discriminate between disulfide bonds and thiol groups and thus provides insight into the structure of **peptide 8**^26^. The ^13^C-NMR spectra reveal that the C_β_ chemical shifts of Cys13 and Cys3 are shifted to lower fields (at 42.07 and 38.84 ppm) as compared to the Cys2 and Cys7 C_β_ shifts that are located at 28.13 and 28.12 ppm. The more downfield shifts of the Cys3 and Cys13 signals are indicative of the formation of a monocyclic peptide that contains a disulfide bridge between Cys3 and Cys13.

**Fig. 5:**
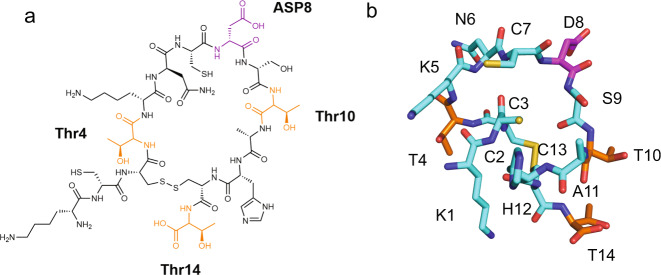
The structure of the ice-binding peptide 8. **a** Chemical structure of **peptide 8**. Threonine residues are highlighted in orange and aspartic acid is coloured in magenta. **b** 3D Representation of **peptide 8** as determined by constrained Rosetta with individual residues labelled. The carbon backbone is shown in cyan, nitrogen is coloured red, oxygen is coloured blue, sulfur is coloured yellow, aspartic acid is coloured in magenta, and threonines are coloured orange.

The chemical structure of cyclic peptide **8** is shown in Fig. 5a. To build a 3D structural model of peptide **8**, constrained CS-Rosetta^27^ was used. Based on the recorded chemical shifts, 1500 model structures were generated, which were first sorted based on their relative energy (Supplementary Fig. 14A). The ten structures with the lowest relative energy were further evaluated by aligning the structures and comparing their relative RMSD values. An overlay of the top ten models is presented in Supplementary Fig. 14B. The model with the lowest relative energy and RMSD value is presented in Fig. 5b. It reveals an unordered, cyclic peptide with a spacing of 4.8 Å between the β-carbons of Thr 10 and Thr 14 side chains. The 4.8 Å distance between Thr residues is interesting as it is a common motif of the ice-binding sites of IBPs found in overwintering organisms including the beetle *Tenebrio molitor*^28^. Supplementary Fig. 14C presents the ten lowest relative energy structures that were generated. The distances between the β-carbons of the Thr10–Thr14 sidechains in the other nine structures were determined to vary between 6.4 and 12.4 Å. In agreement with the short length and cyclic structure of **peptide 8**, both circular dichroism spectroscopy (supplementary fig. 15) as well as NMR chemical shift analysis (supplementary Fig. 16) provided relatively little evidence for the presence of any ordered secondary structure. Diffusion ordered spectroscopy NMR experiments, which were carried out with 1 mM solutions of **peptide 8** in 0.5× PBS (pH = 6), and small-angle X-ray scattering (SAXS) analysis of 1 mM solutions of **peptide 8** in 1× PBS pointed toward the existence of a single species and the absence of any higher order, aggregated assemblies under these conditions (Supplementary Figs. 17 and 18).

### Mutational analysis of peptide activity

**Fig. 6:**
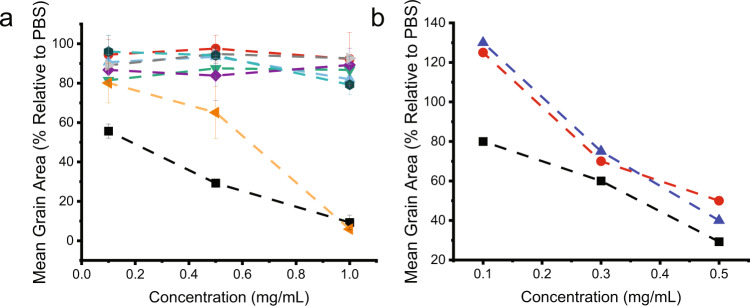
Ice recrystallization inhibition activity of peptides listed in Fig. 7. **a** Mean grain area analysis of IRI activity of the peptides listed in Fig. 7. All IRI assays were performed in oxidizing conditions unless otherwise noted. Black squares represent the oxidized **peptide 8**. The red circle is reduced **peptide 8**. Orange triangles are Thr4-Ser mutation. Purple diamonds are Thr10-Ser mutation. Green triangles are Thr14-Ser mutation. Blue triangles all Thr have been mutated to Ser. Grey triangles is a scrambled peptide sequence. Teal hexagons Asp8-ser mutation. (values reported are averaged from three individual experiments and shown as mean data values ± SD. Error bars represent standard deviation). **b** Mean grain area IRI activity comparison of **peptide 8** (black squares) to literature reported values for polyvinyl alcohol (PVA56) (red circles) and a truncated type I antifreeze protein 22 amino acids in length modified with anthracene (PC-AFP_22_ monomer) (blue triangles)^29^.

Dose-dependent IRI activity studies indicated that the cyclic structure of **peptide 8** is essential for its activity. This is illustrated in Fig. 6a, which compares the activity of **peptide 8** under reducing (red circle) and oxidizing (black square) conditions. As can be seen, the peptide is only active when it is oxidized, suggesting the cyclization is essential. The activity of **peptide 8** is comparable to that of other IRI active compounds such as PVA56 (red circles) and truncated type I AFP (blue triangles) (Fig. 6b) in spite of the smaller molecular weight of **peptide 8** as compared to these two compounds^29^. This is an interesting observation as ice-binding activity is related to size, with larger molecules typically being more active than smaller molecules^30,31^.

**Fig. 7:**
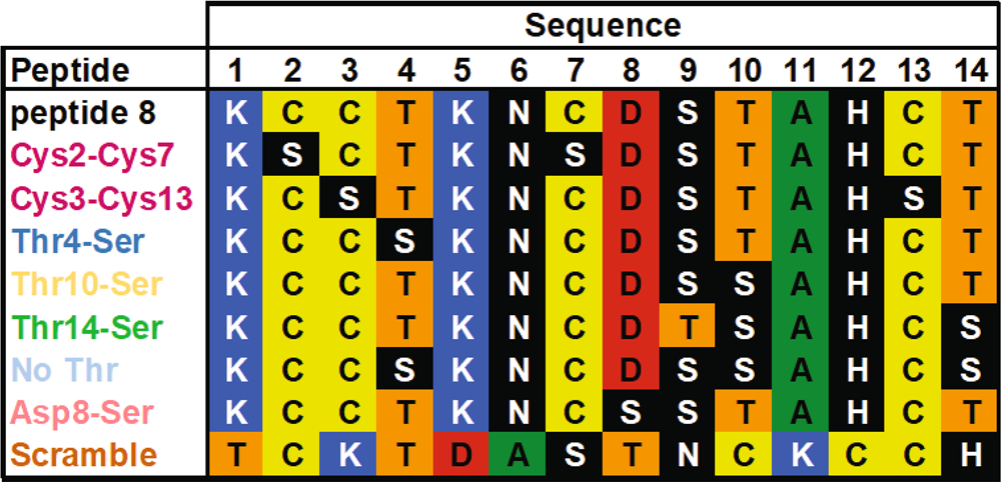
Peptides studied for ice recrystallization inhibition activity. Listed are the sequence of **peptide 8** as well as of the mutants of **peptide 8**, which were investigated to establish structure-activity relationships.

To underpin the importance of the cyclic structure of **peptide 8**, two mutants were prepared in which several cysteine residues were replaced by serine (Fig. 7, HPLC and MS spectra are provided in Supplementary Figs. 19 and 20). Mutating Cys2 and Cys7 to serine had no impact on IRI activity at 1 mg/mL, whereas changing Cys3 and Cys13 to serine abolished the activity (supplementary fig. 21). Taken together, these data indicate that cyclization through Cys3 and Cys13 as determined by NMR is essential for activity.

In order to obtain further insight into the structure–activity relationships that govern the activity of **peptide 8**, additional mutant peptides were synthesized with the threonine residues, which are essential to the activity of many IBPs, mutated for a serine (Fig. 7)^32^. A total of four mutants was prepared in which either threonine 4, threonine 10 or threonine 14 or all three threonine residues were replaced by serine. HPLC and LS–MS data for the mutant peptides are provided in Supplementary Figs. 22–25. Figure 6a compares the dose-dependent IRI activity of **peptide 8** with that of the different mutant peptides (results covering an expanded range of concentrations are shown in Supplementary Fig. 26). Interestingly, mutating Thr4 (orange triangles) to serine did not affect the IRI activity at 1 mg/mL, indicating it is not required for activity. However, mutating Thr10 (purple diamonds) or Thr14 (green triangles) resulted in a complete loss of activity, signalling they are involved in the IRI activity. In addition, mutating all Thr to Ser (blue triangles) concurrently, resulted in a total activity loss highlighting the functional importance of the threonine residues. Furthermore, due to the proximity of aspartic acid residue 8 (Asp8) to Thr10 in the solution structure of **peptide 8**, it was also examined by mutational analysis for impact on activity (HPLC and MS spectra of this peptide are presented in supplementary fig. 27). As Fig. 6 indicates, mutating Asp8 to Ser (teal hexagons) led to a complete loss of activity, indicating it is involved with ice binding. Finally, a randomized peptide sequence was synthesized as a control peptide (HPLC and MS spectra Supplementary Fig. 28). This scrambled peptide also did not reveal any IRI activity (Fig. 6A, grey triangles) highlighting the sequence-selectivity of the ice-binding properties of **peptide 8**. These observations illustrate the importance of three residues, which play a key role in anchoring **peptide 8** to ice and preventing ice recrystallization, thus bringing new insight into what are essential structural features.

### Molecular dynamics simulations

**Fig. 8:**
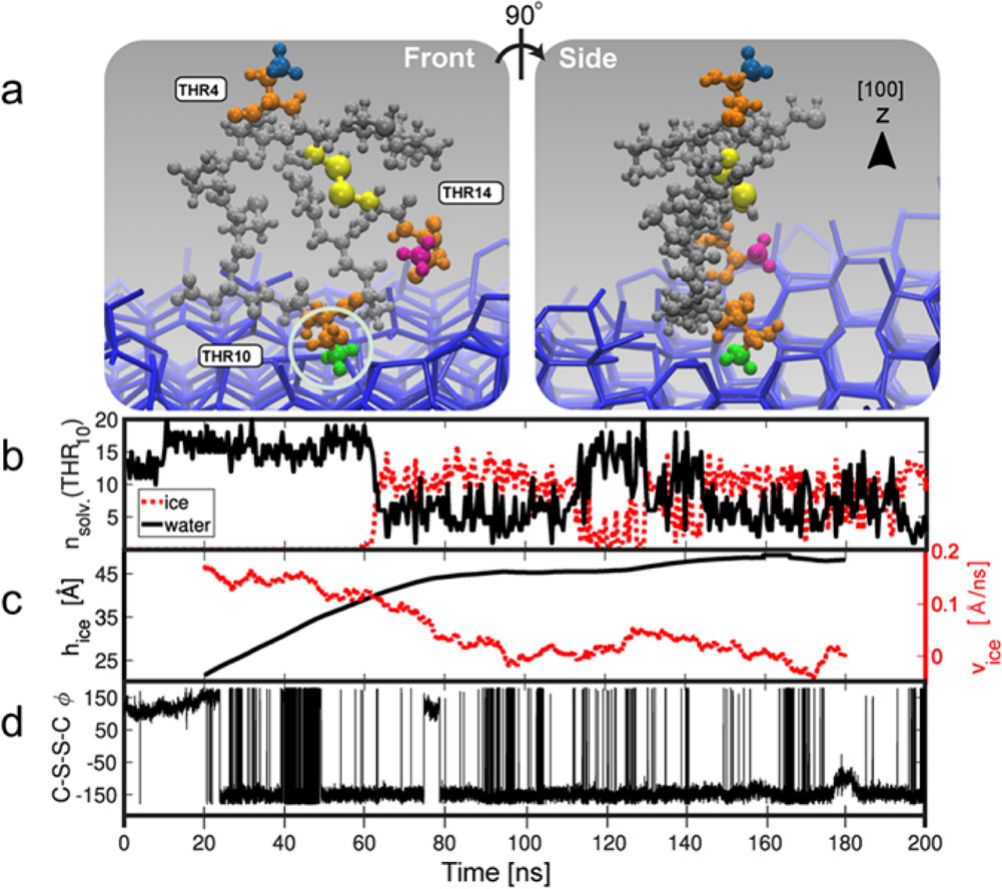
Molecular dynamics simulations of peptide. **a** Snapshots of **peptide 8** upon binding to ice from two different perspectives. The Thr residues are coloured orange, and their respective CH_3_ groups are as follows; green is CH_3_ on Thr10, blue is CH_3_ on Thr4 and magenta is CH_3_ on Thr14. The disulfide bond torsional angle (*φ*) is coloured yellow. Adsorption of CH_3_ to a growing ice front, the methyl fits into a pocket on the primary prismatic front (circled). **b** Representation of the amount of liquid (black line) and ice-like (red line) water in the solvation shell of Thr10. **c** Plot of ice growth in Angstroms and corresponding growth rate Angstrom/nanosecond. Adsorption of the peptide at 60 ns, slows down the growth of the ice front in the *z* direction (black line) and reduces growth rate (dashed red line). **d** Analysis of the C–S–S–C torsional angle, *φ*, around the disulfide bond throughout the simulation; Note that due to the use of a 5000 point moving average, the first and last 20 ns have been omitted. (Snapshots are from trajectory 18 in Supplementary Fig. 29).

The molecular-level details of the interaction between **peptide 8** and ice were further investigated with MD simulations using the solution structure of the peptide (obtained via NMR and CS-Rosetta) as a starting point. Using CHARMM36^33^ and the TIP4P/Ice^34^ force fields for the peptide and water molecules, respectively, a total of 20 independent unbiased simulations were performed. The simulations were run at 265 K and for 200 ns each. Trajectory analyses plotting the number of ice-like and liquid water molecules around the methyl group of the Thr10 residue for each of the 20 independent MD simulations can be found in Supplementary Fig. 29. At the start of the simulations, **peptide 8** was placed in a random orientation situated away from the growing ice front. Figure 8a illustrates a snapshot of **peptide 8** from two different perspectives upon binding to ice. The snapshots in Fig. 8a are taken from Trajectory 18 in Supplementary Fig. 29 at 70 ns. The simulations reveal that the binding activity of the peptide to ice is due to hydrophobic interactions of the methyl group on Thr10. This is highlighted in Fig. 8a, which shows a (de-solvated) methyl group on Thr10 fitting into a cavity of the [100] ice surface. Figure 8b indicates that, on average, the methyl group is surrounded by 16 liquid water molecules, as seen in the first 60 ns of the simulation during which time the peptide is diffusing through the bulk solution. Upon binding of the peptide to ice, some of the water molecules leave the methyl solvation shell, being in part replaced by the water molecules found in the ice front. This de-solvation process is illustrated in Fig. 8b, where a change in the water phase is observed for the solvation shell at around 60 ns, the time at which **peptide 8** starts to bind. Once **peptide 8** has bound to ice, further ice growth is substantially hindered, as shown in Fig. 8c. The simulations indicate that binding of **peptide 8** to ice is predominantly entropically driven by the hydrophobic de-solvation of the methyl groups, which in turn is strongly influenced by the conformational space the peptide can occupy. Analysis of the C–S–S–C torsional angle, *φ*, around the disulfide bond (highlighted in yellow in Fig. 8a) indicates that **peptide 8** can assume two different conformations characterized by negative or positive values of *φ* (see Fig. 8D and Supplementary Fig. 29). Supplementary Fig. 30 shows contour plots that present the number of water molecules around Thr10 as a function of the C–S–S–C torsional angle. These plots compare the number of water molecules in the solvation shell around the Thr10 methyl group along the entire MD trajectory until **peptide 8** starts interacting with ice with that found within the restricted time section (5 ns) before the peptide binds with ice. This analysis reveals that in particular in the short time frame prior to ice binding there is an increase in the number of water molecules within the solvation shell of Thr10 methyl groups. This suggests that the ability of **peptide 8** to interact with ice appears to be enhanced by specific conformations, which maximize the amount of water molecules within the solvation shell of Thr10. As a whole, these findings unequivocally underline the critical role of Thr10 in binding of **peptide 8** to ice. In contrast, the contributions of Thr14 and Asp8, which were also identified via mutational analysis as essential residues, are less clear. Analysis of the hydrogen bonding interactions between **peptide 8** and ice (supplementary fig. 31) suggests additional hydrogen bonding between Asp 8 and ice, along with further hydrophobic binding between Thr14 and ice. Together, these supplemental interactions may further stabilize the binding of Thr10 with ice.

In many cases, IBPs interact with ice by means of a layer of clathrate-like water molecules (the so-called “anchored clathrate waters” mechanism)^35^. To probe the presence of clathrate water around **peptide 8**, 20 statistically independent MD trajectories were analyzed. These analyses, however, did not reveal the emergence of such layer as shown in Supplementary Fig. 32, which presents a quantitative measure of clathrate-like water around the Thr residues. The absence of clathrate water may be attributed to the small size and unordered solution structure of the peptide, which is in contrast to the well-defined tertiary and quaternary structure of conventional IBPs^1^.

The MD simulations were further used to analyze the intermolecular Thr–Thr distances in **peptide 8**. Supplementary Fig. 33 presents probability density functions of the various intermolecular Thr–Thr distances (measured between the amino acid side chain β-carbons) during the time prior to binding of **peptide 8** to ice with the intermolecular distances during ice binding. Qualitatively, the distribution of distances that is observed corresponds fairly well with the distances that were obtained from the solution NMR analysis (Supplementary Fig. 14). Comparison of Supplementary Fig. 33A, B also indicates that ice-binding results in an increasing probability of peptide conformations that are characterized by short Thr10–Thr 14 distances. Finally, RMSD analysis of the peptide indicates it is flexible and relatively dynamic (Supplementary Fig. 34).

### “Ice-tag” affinity purification

**Fig. 9:**
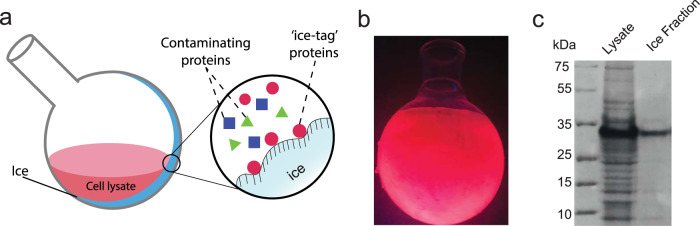
‘Ice-tag’ ice-affinity purification of mCherry. **a** Schematic representation of ‘ice-tag’ ice-affinity purification. **b** Ice shell imaged under UV light. **c** SDS-PAGE of *E. coli* cell lysate containing overexpressed mCherry and fraction that bound ice from the ice-affinity purification. Experiments were performed in triplicate.

The ice-binding properties of **peptide 8** pave the way for a variety of possible applications. One original use of **peptide 8** would be as a fusion sequence to enable “ice-tag” affinity purification of proteins (Fig. 9a and Supplementary Fig. 35). In a first proof-of-concept experiment, **peptide 8** was expressed in *E. coli* bacterial cell culture as a fusion (‘Ice-Tag’) at the C-terminal end of mCherry. The mCherry-**peptide 8** fusion was isolated from the bacterial cell lysate via a single ice-affinity purification step. To this end, the crude bacterial cell lysate was added to a round bottom flask that was covered with a preformed, thin layer of ice. After 15 min, the remaining liquid was decanted. This resulted in the formation of a scarlet coloured ice shell within the flask (Fig. 9b). SDS-PAGE analysis of the molten ice-fraction revealed the presence of a single, prominent band with a mass between 25 and 35 kDa, illustrating the efficiency of the ice-tag purification (Fig. 9c). The identity of the protein captured by the ice-affinity layer was confirmed by MALDI-TOF mass spectrometry, which indicated a single peak with the expected mass as determined by MALDI-TOF (Expected mass 28.4 kDa compared to measure mass 28.3 kDa, Supplementary Fig. 35C). To further evaluate the efficiency of **peptide 8** as an ice-affinity tag, rotary ice-purification of *E. coli* cell lysate expressing mCherry modified with a C-terminal **peptide 8** tag was compared with that of *E. coli* cell lysate containing overexpressed GFP, which shares high sequence and size similarity to mCherry, without an ice-tag. The total protein content in the cell lysate, as well as in the ice phase and in the liquid phase was assessed by the BCA assay as well as via fluorescence spectroscopy. Under UV-irradiation, the ice shell of the flask used for the GFP “purification” showed very little fluorescence as compared to the ice shell that was used to capture the **peptide** **8**-tagged mCherry (Supplementary Fig. 36A). Analysis of the protein content in the liquid fraction and the ice fraction showed that ice purification of the **peptide 8**-tagged mCherry lysate resulted in isolation of ~50% of the total protein content, as compared to just ~20% in case of the GFP containing control lysate (Supplementary Fig. 36B, C). More importantly, SDS-PAGE analysis indicated that “ice tag” purification of **peptide 8**-modified mCherry was accompanied by an increase in protein purity, whereas the control experiment with the GFP lysate showed no improvement in protein purity (Supplementary Fig. 36D).

## Discussion

Phage display was successfully used to identify a 14 amino acid, cyclic ice-binding peptide. By combining ice-binding activity studies with NMR and modelling tools, it was established that the peptide has a cyclic structure and features three residues essential for ice binding. The strategy presented here complements traditional de novo design approaches to ice-binding peptides. Since the phage display ice-affinity selection process starts from a randomized peptide library, a vast diversity space can be screened for potential ice-binding peptides. The short peptides that can be identified with the help of phage display may help to provide further structural insight into the key structural determinants that govern the properties of IBPs. Furthermore, since phage display generally leads to short peptides that are composed of naturally occurring amino acids, the strategy reported here may also enhance the synthetic accessibility of novel ice-binding peptides.

### Reporting summary

Further information on research design is available in the Nature Research Reporting Summary linked to this article.

## Supplementary information

Supplementary Information

Reporting Summary

## Data Availability

The data that support the findings of this study are available from the corresponding author upon reasonable request.
