# Supplementary material for: A minimalistic cyclic ice-binding peptide from phage display
Source: Nat Commun. 2021 May 11;12:2675. doi: 10.1038/s41467-021-22883-w (PMC8113477; doi:10.1038/s41467-021-22883-w)
Supplement: Supplementary file 1 — Supplementary Information [file 41467_2021_22883_MOESM1_ESM.pdf]

Supplementary Information

A Minimalistic Cyclic Ice-Binding Peptide from Phage Display

Corey A. Stevens[1], Fabienne Bachtiger[2], Xu-Dong Kong[3], Luciano A. Abriata[4], Gabriele C. Sosso[2], Matthew I. Gibson[5,6], Harm-Anton Klok[1,*]

1. *Laboratoire des Polymères, Institut des Matériaux and Institut des Sciences et Ingénierie Chimiques, École Polytechnique Fédérale de Lausanne (EPFL), Batiment MXD, Station 12, 1015 Lausanne, Switzerland*
2. *Department of Chemistry and Centre for Scientific Computing, University of Warwick, Gibbet Hill Road, Coventry CV4 7AL, United Kingdom*
3. *Laboratory of Therapeutic Proteins and Peptides, Institute of Chemical Sciences and Engineering, École Polytechnique Fédérale de Lausanne (EPFL), Batiment CH, Forel 2, 1015 Lausanne, Switzerland*
4. *Protein Production and Structure Core Facility and Laboratory for Biomolecular Modeling, École Polytechnique Fédérale de Lausanne (EPFL) and Swiss Institute of Bioinformatics, Batiment AI, Station 19, 1015 Lausanne, Switzerland*
5. *Department of Chemistry, University of Warwick, Gibbet Hill Road, Coventry CV4 7AL, United Kingdom*
6. *Warwick Medical School, University of Warwick, Gibbet Hill Road, Coventry CV4 7AL, United Kingdom*

* Corresponding author: harm-anton.klok@epfl.ch

Supplementary Figures

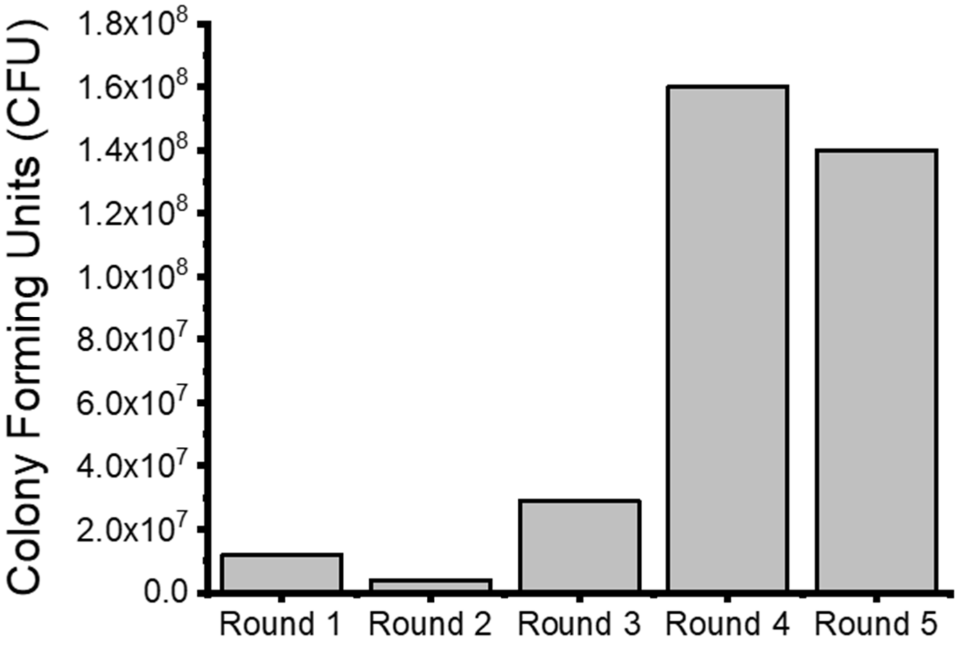

Supplementary figure 1. Evolution of phage titer with increasing rounds of ice-affinity selection. Titers were calculated by infecting *E. coli* with phages eluted after each round of selection and plating on 2×YT-Agar + tetracycline plates, resulting in colonies that were counted.

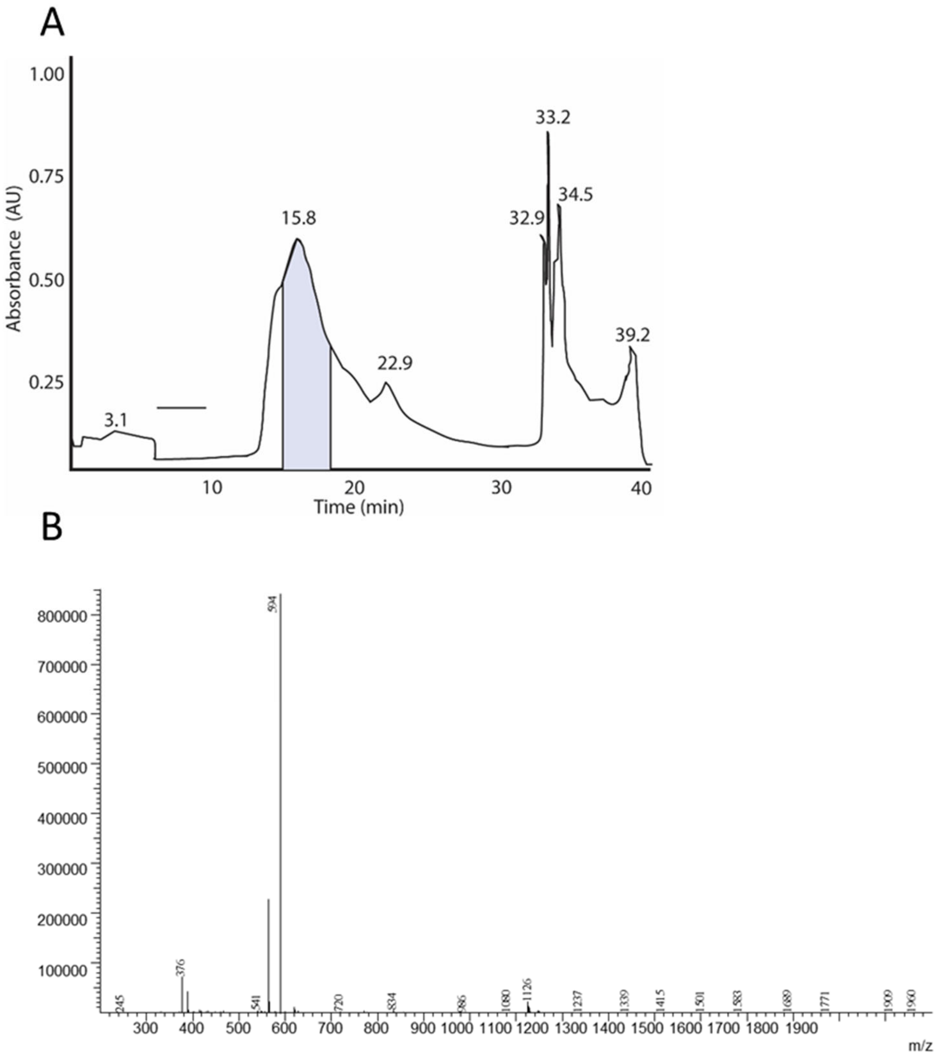

Supplementary figure 2. HPLC and LC-MS data of peptide 1. a Preparative HPLC of **peptide 1** (clones 64-66 in supplementary table 1: sequence KCCTESPLCCT). Highlighted in light purple is the product peak that was collected and examined by LC-MS and checked for IRI activity. **b** LC-MS data of the peptide.

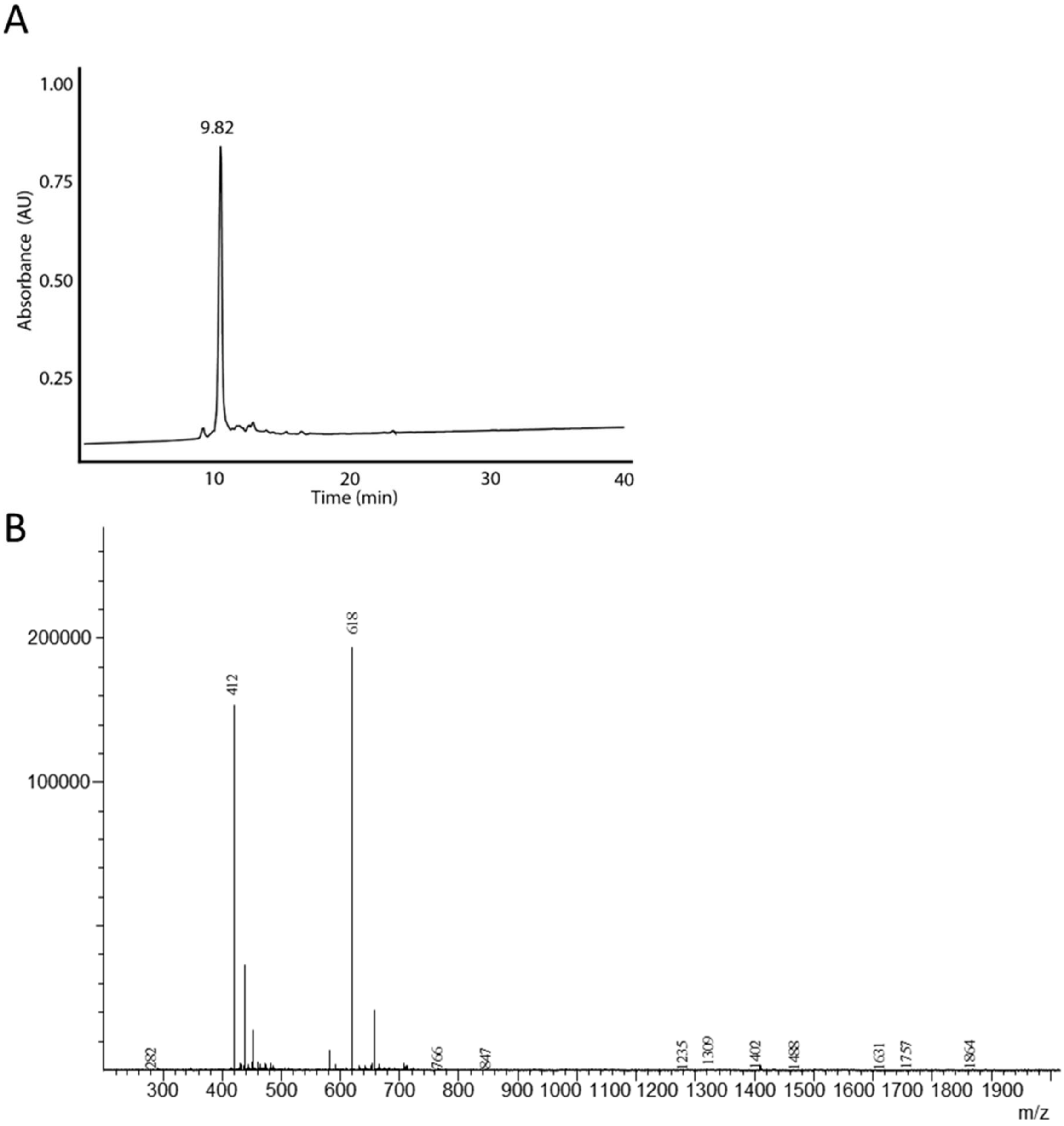

Supplementary figure 3. HPLC and LC-MS data of peptide 2. a Preparative HPLC of **peptide 2** (clones 23-27 in supplementary table 1: sequence GCVSCCQGTCF). **b** LC-MS data of the peptide.

A

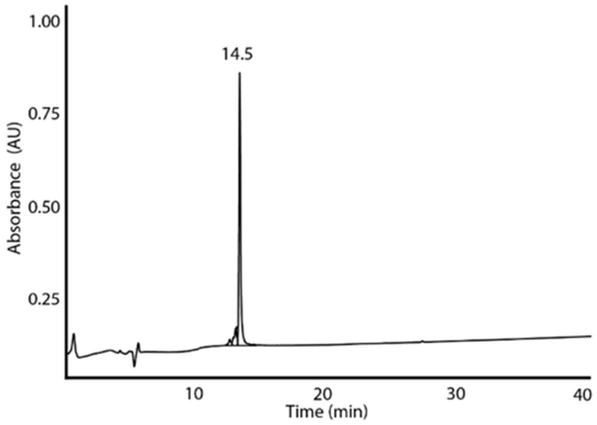

B

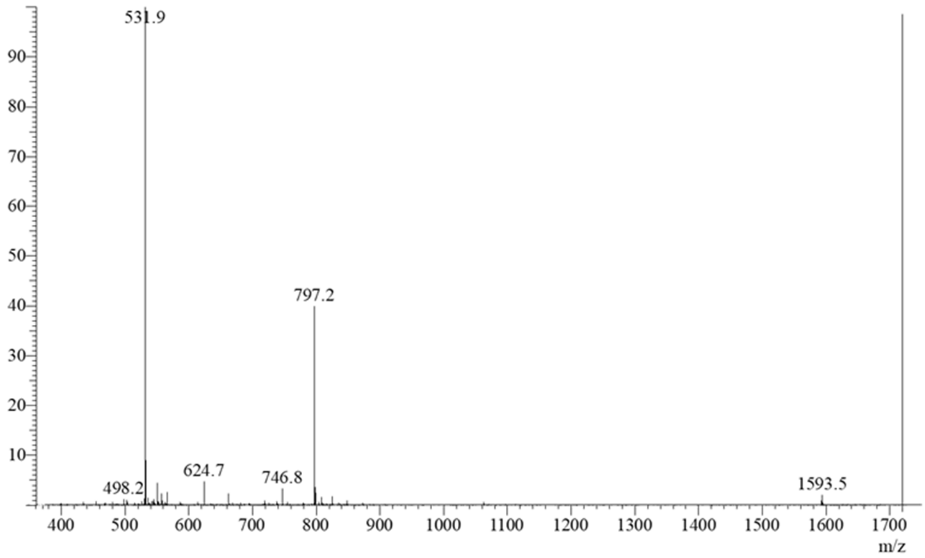

Supplementary figure 4: HPLC and LC-MS data of peptide 3. a Preparative HPLC of **peptide 3** (clones 10-14 in supplementary table 1: sequence TCSRTHQCTRPCCP). **b** LC-MS data of the peptide.

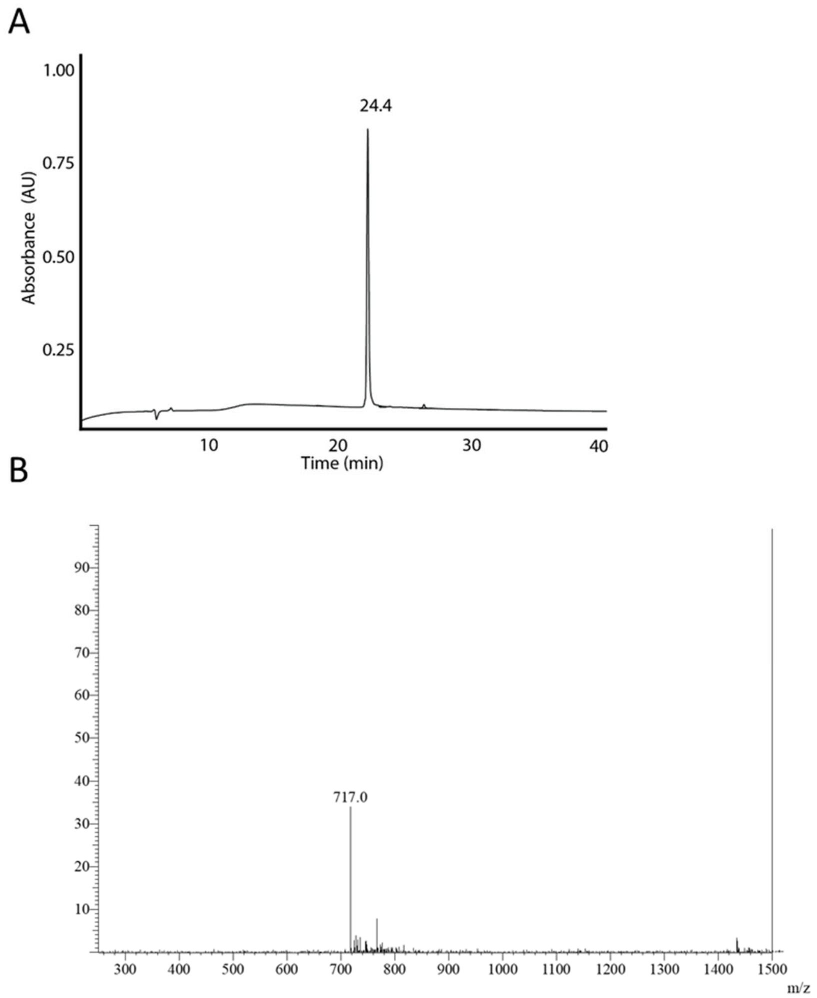

Supplementary figure 5. HPLC and LC-MS data of peptide 4. a Preparative HPLC of **peptide 4** (clones 4-9 in supplementary table 1: sequence SCSTCNDSVSDCCL). **b** LC-MS data of the peptide.

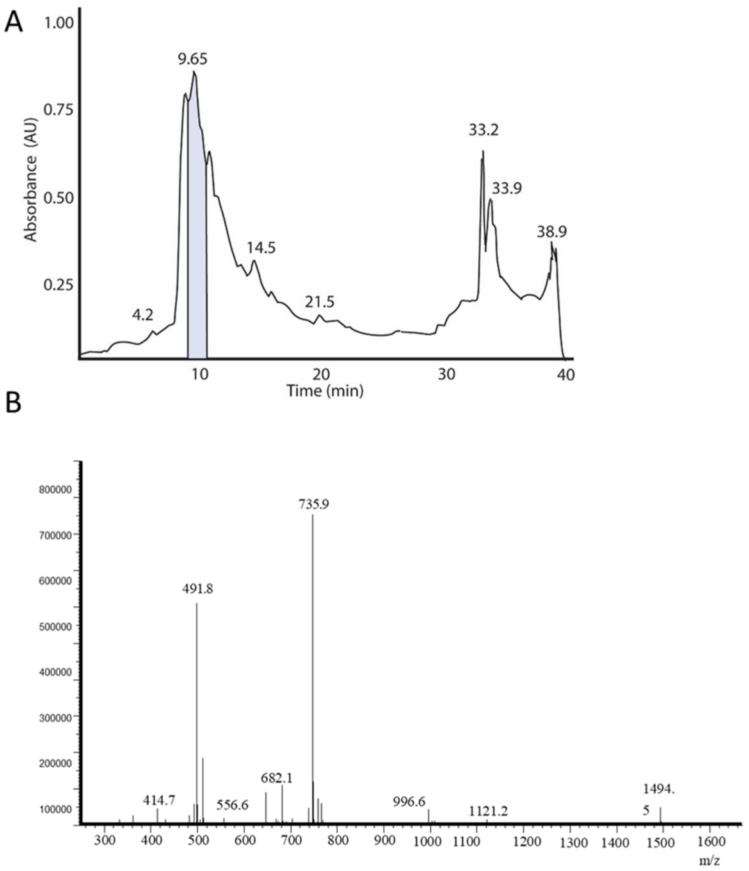

Supplementary figure 6. HPLC and LC-MS data of peptide 5. a Preparative HPLC of **peptide 5** (clones 83-88 in in supplementary table 1: sequence QCMRQPCENCCR). Highlighted in light purple is the product peak that was collected and examined by LC-MS and checked for IRI activity. **b** LC-MS data of the peptide.

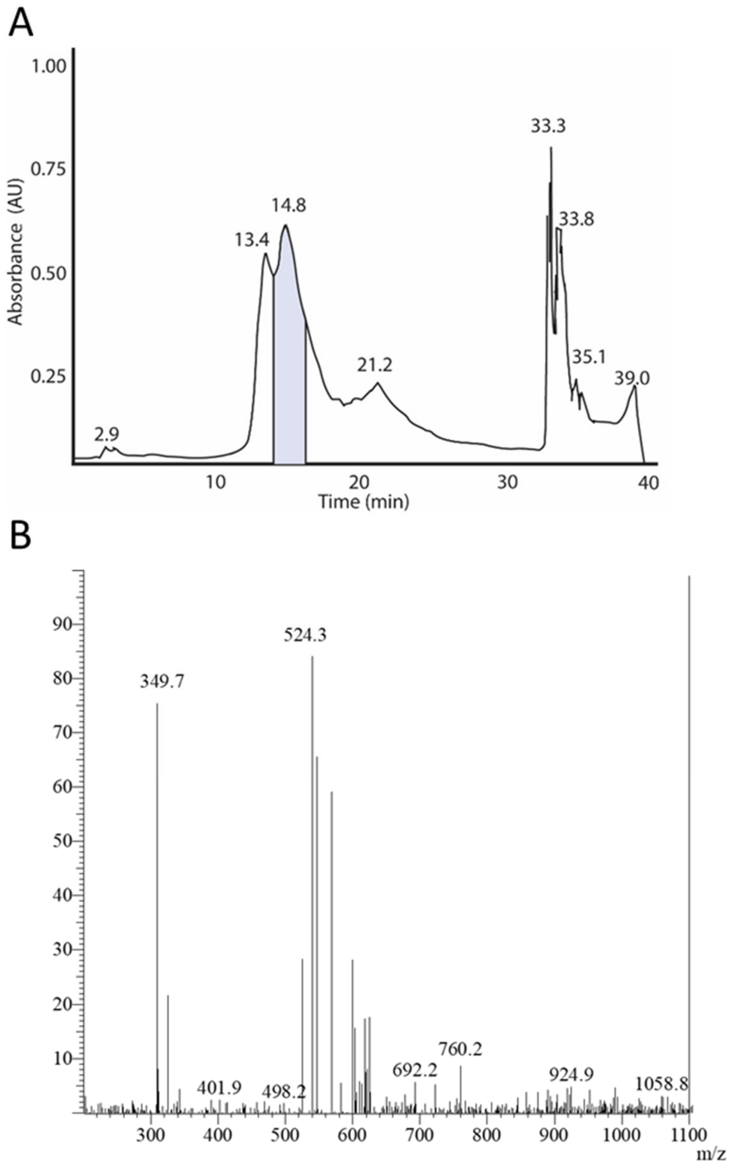

Supplementary figure 7. HPLC and LC-MS data of peptide 6. a Preparative HPLC of **peptide 6** (clones 15-21 in supplementary table 1: sequence TCCAFCVSCI). Highlighted in light purple is the product peak that was collected and examined by LC-MS and checked for IRI activity. **b** LC-MS data of the peptide.

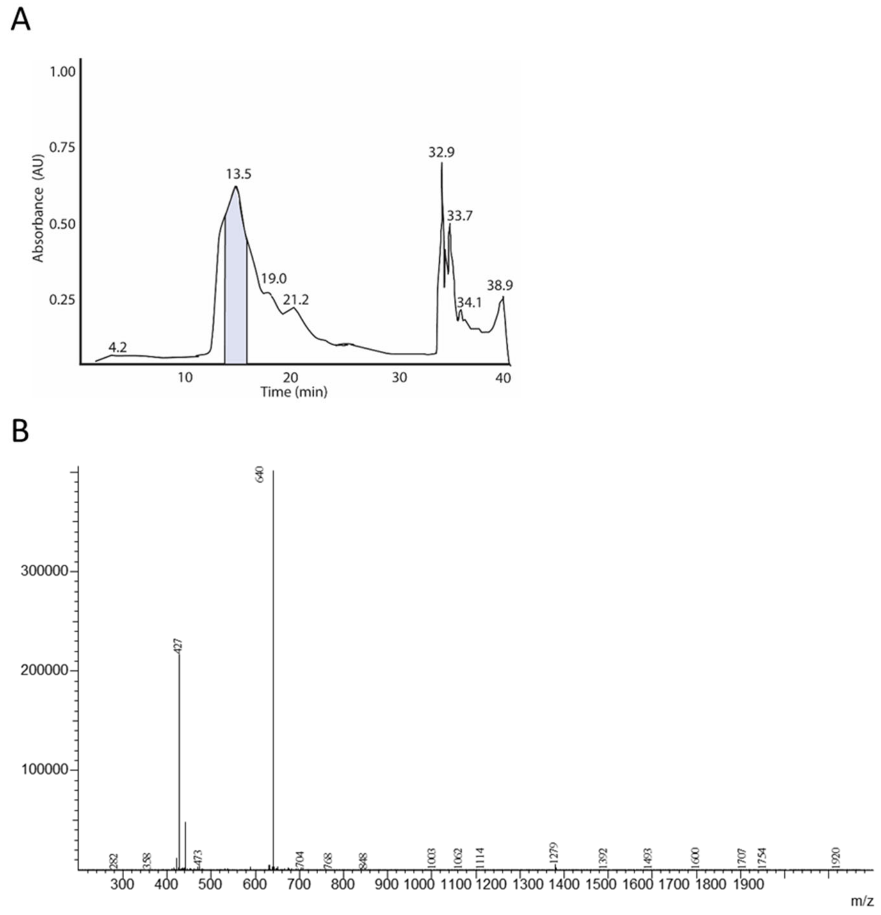

Supplementary figure 8. HPLC and LC-MS data of peptide 7. a Preparative HPLC of **peptide 7** (clones 45-52 in in supplementary table 1: sequence RCDHCLCLQCS). Highlighted in light purple is the product peak that was collected and examined by LC-MS and checked for IRI activity. **b** LC-MS data of the peptide.

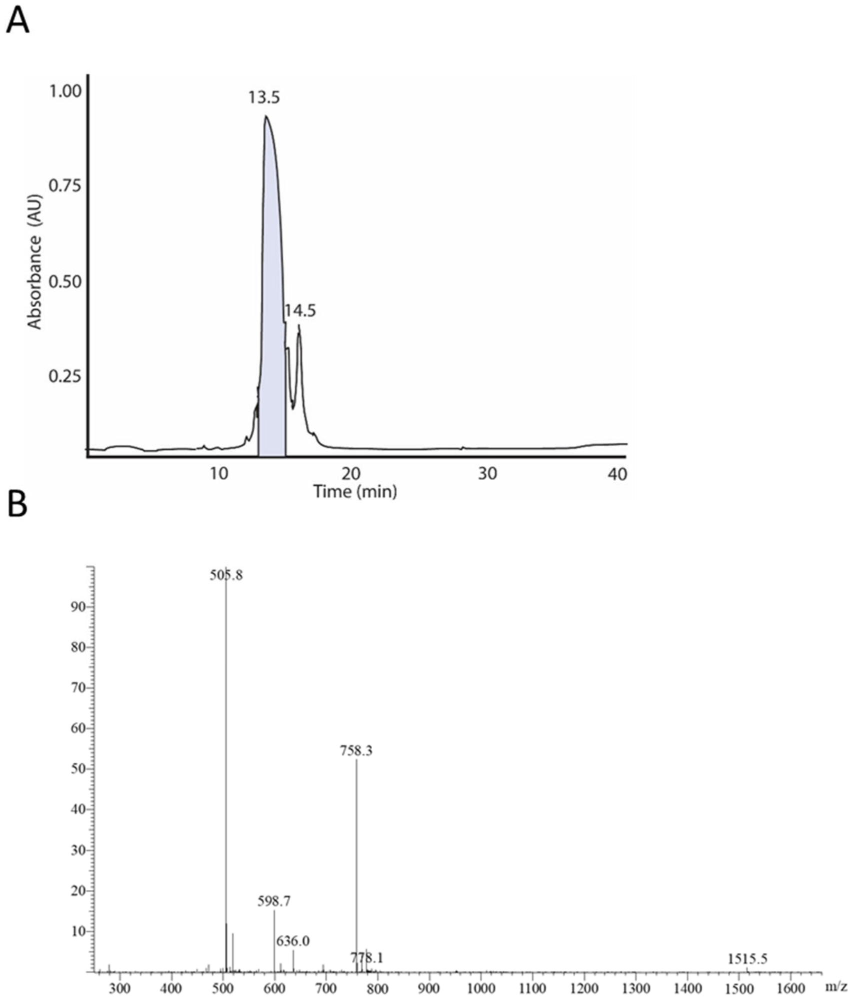

Supplementary figure 9. HPLC and LC-MS data of peptide 8. a Preparative HPLC of **peptide 8** (clones 55-63 in supplementary table 1: sequence KCCTKNCDSTAHCT). Highlighted in light purple is the product peak that was collected and examined by LC-MS and checked for IRI activity. **b** LC-MS data of the peptide.

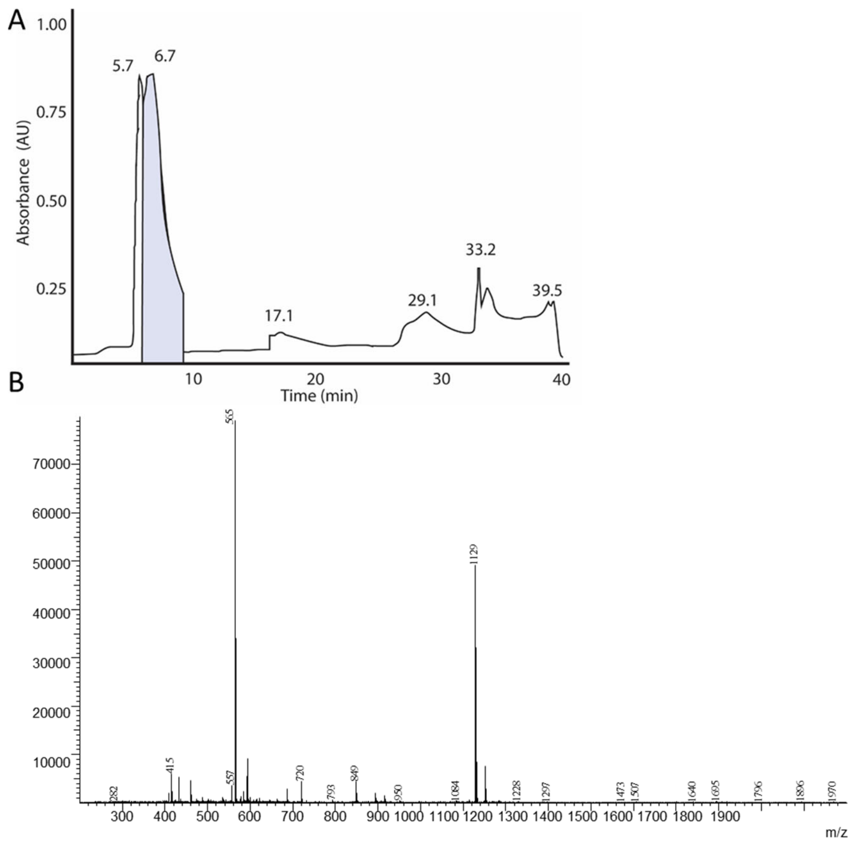

Supplementary figure 10. HPLC and LC-MS data of peptide 9. a Preparative HPLC of **peptide 9** (clones 67-77 in in supplementary table 1: sequence KCCNVINCCK). Highlighted in light purple is the product peak that was collected and examined by LC-MS and checked for IRI activity. **b** LC-MS data of the peptide.

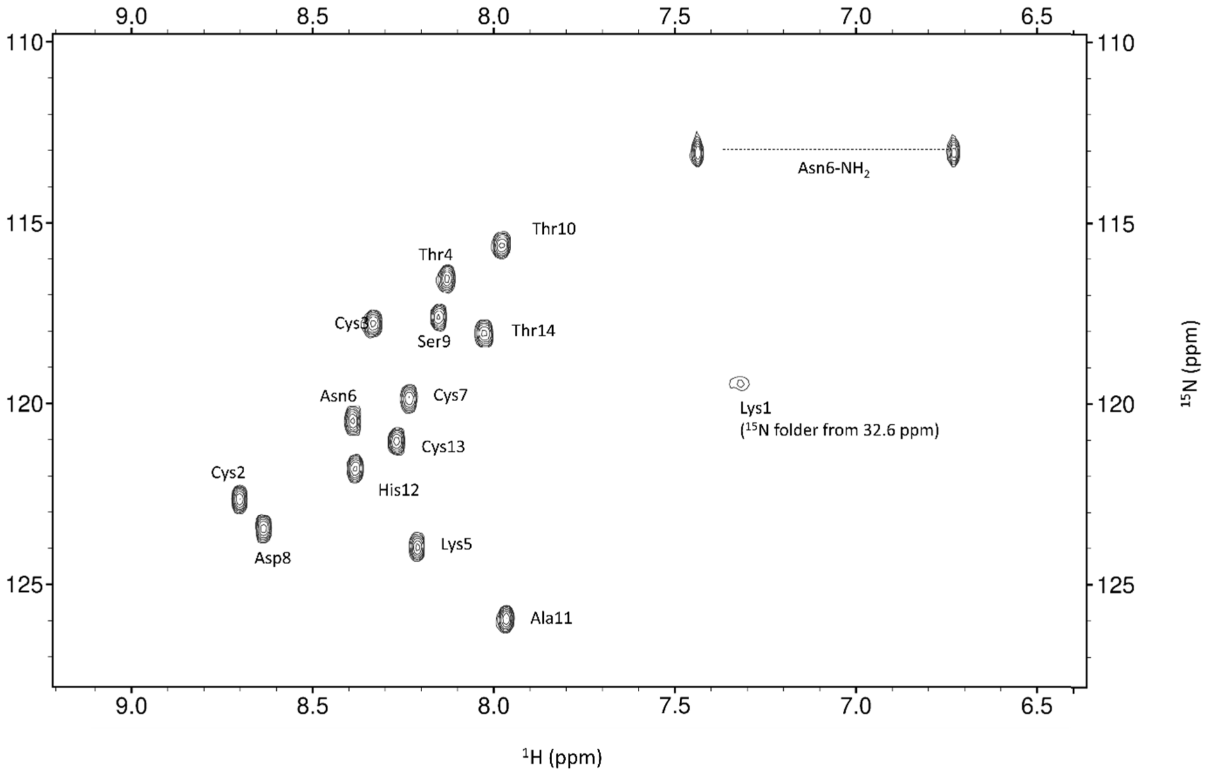

Supplementary figure 11. ^{1}H, ^{15}N HSQC NMR spectrum of peptide 8 obtained at natural ^{15}N abundance.

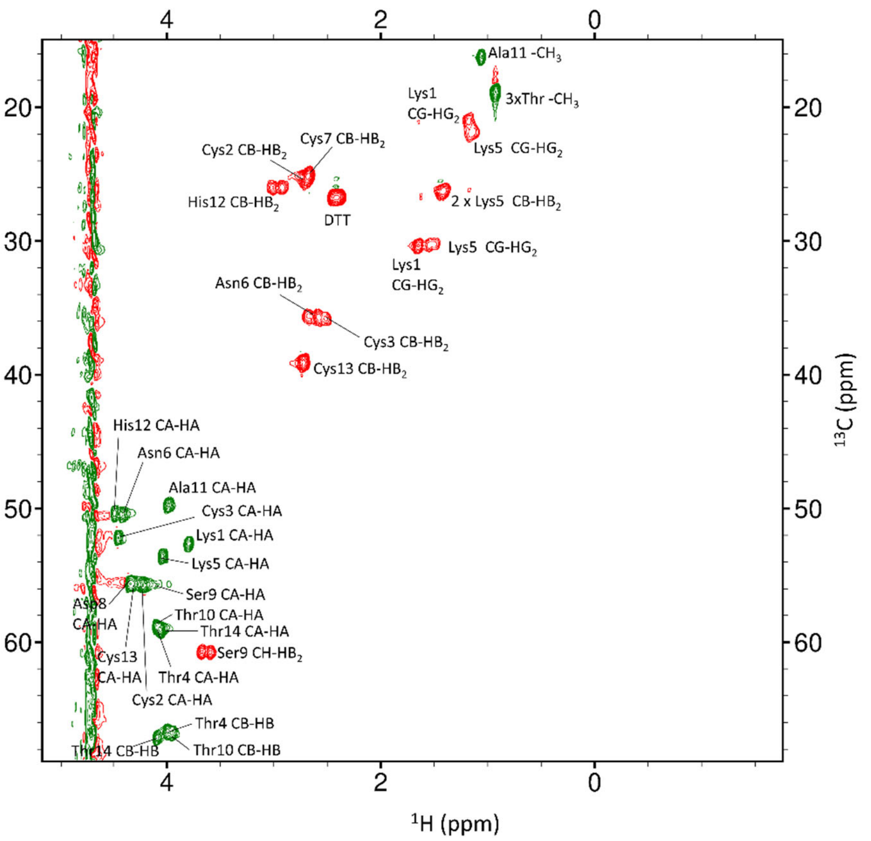

Supplementary figure 12. Multiplicity-edited ^{1}H, ^{13}C HSQC NMR spectrum of peptide 8. Carbons bound to a single or three protons (CA-HA, threonine CB-HB and methyl C-H) correlations are edited negative and displayed in green, while C-H_2 groups are edited positive and shown in red.

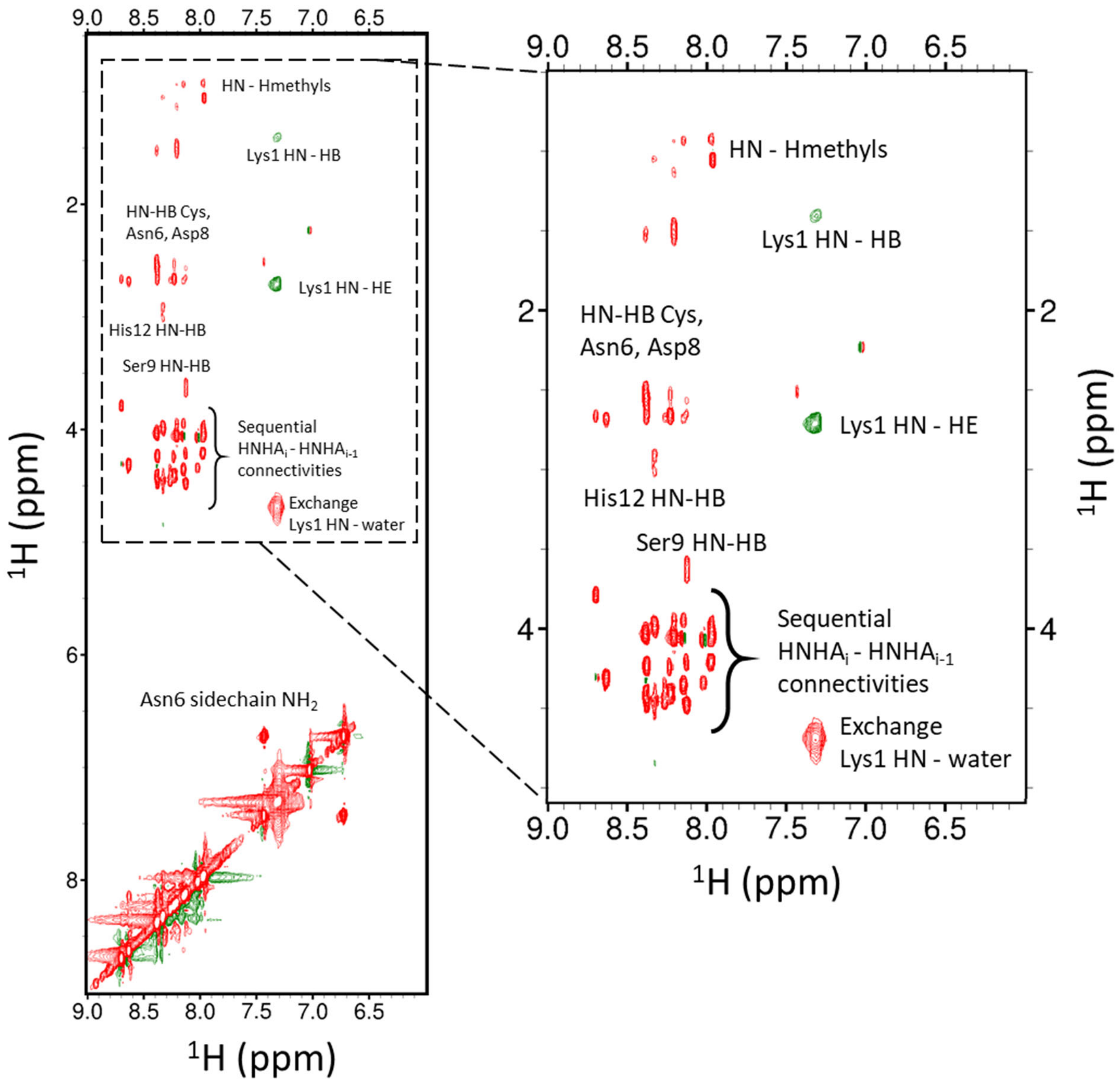

Supplementary figure 13. Key regions of a ^{1}H-^{1}H NOESY NMR spectrum of peptide 8 collected in 10% D_2O with 200 ms of mixing time. Red contours are positive, green are negative. Selected assignments are shown.

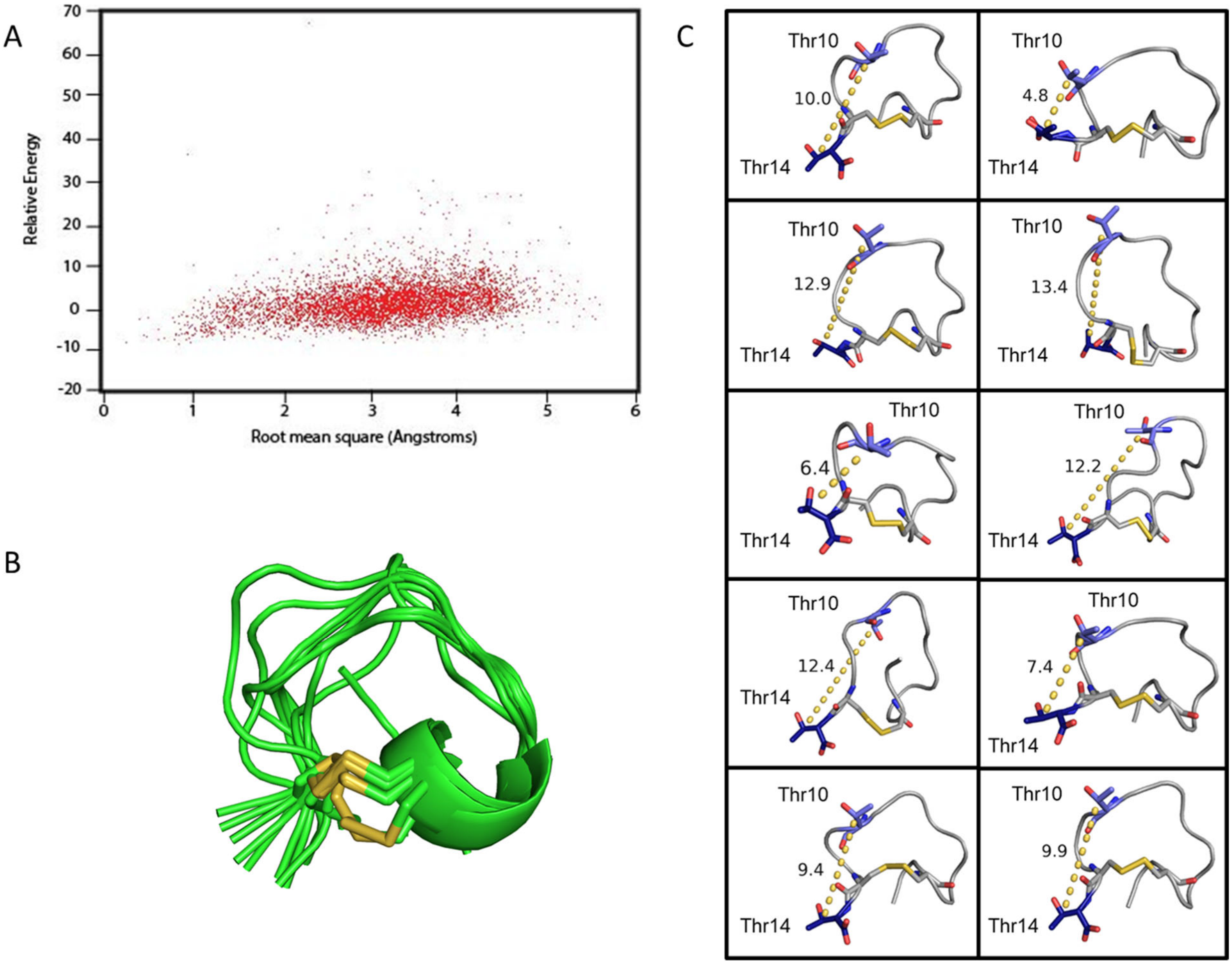

Supplementary figure 14. CS-Rosetta analysis of peptide 8. a Overview of 1500 possible structures obtained for **peptide 8** by CS-Rosetta prediction ranked by lowest relative energy and RMSD deviation. **b** Overlay of the top-10 structures obtained from CS-Rosetta prediction with stick representation of the disulfide bond. Sulphur is coloured yellow. **c** The Thr10 (light blue) – Thr14 (dark blue) spacing in the top-10 structures obtained from CS-Rosetta prediction with stick representations of the Thr residues and the disulfide bond. Sulphur is coloured yellow, oxygen is coloured in red, peptide backbone is coloured in grey. Distances were measured in angstroms between the beta-carbon atoms of the Thr sidechains.

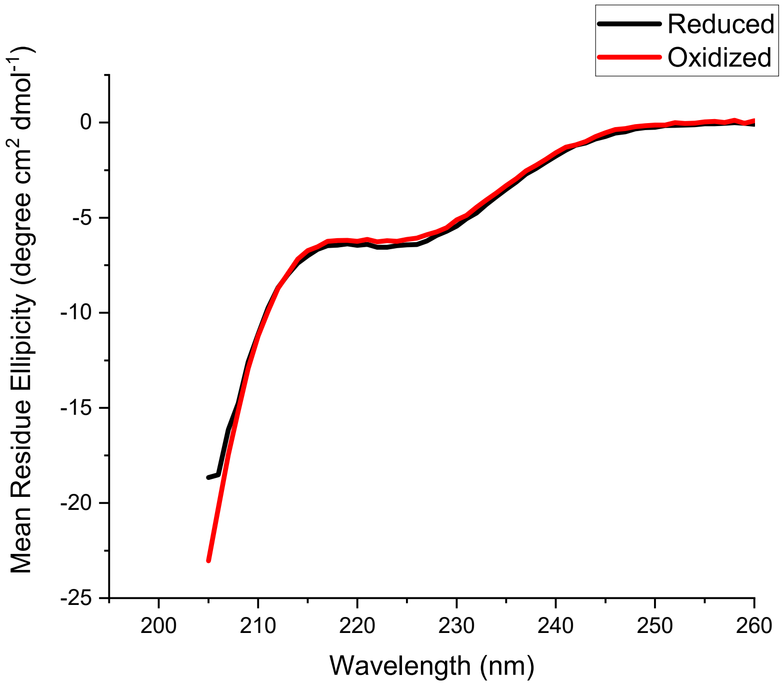

Supplementary figure 15. Circular dichroism spectroscopy analysis of peptide 8. Shown are spectra recorded under reduced (**black**) and oxidized (red) conditions. **Peptide 8** was dissolved to a final concentration of 1 mM in 1 X PBS and the peptide was reduced by adding 10 mM DTT to the solution.

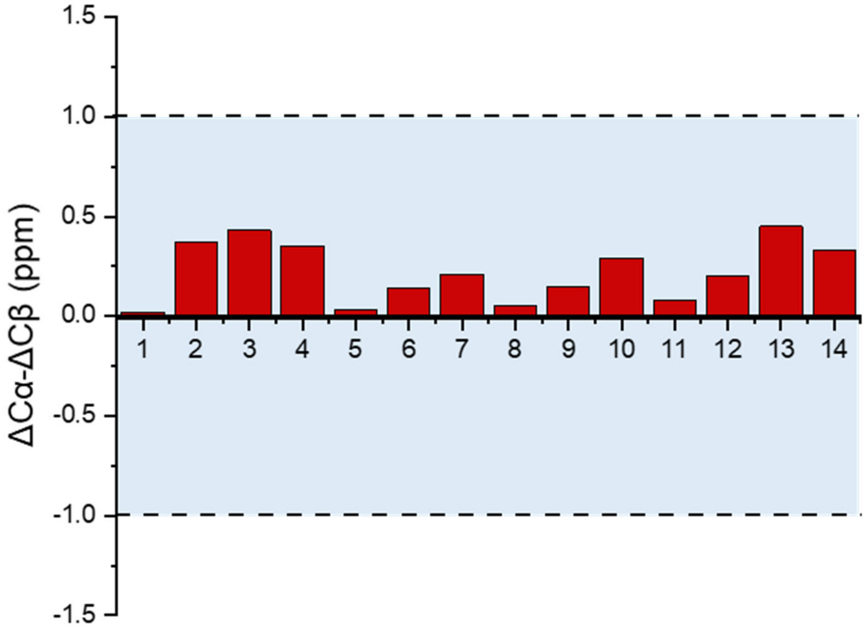

Supplementary figure 16. Residue-specific secondary structure calculation for peptide 8 based on the chemical shift index analysis[27]. ΔCA - ΔCB = ($CA_{measured}$ - $CA_{random\ coil}$) - ($CB_{measured}$ - $CB_{random\ coil}$) is a metric for secondary structure unaffected by calibration and other offsets. Residues with values ≥ 1 indicates alpha-helical secondary structure and residues with values ≤ -1 adopt beta-sheet secondary structure. Residues with values between 1 and -1 are random coil with slight alpha or beta propensity.

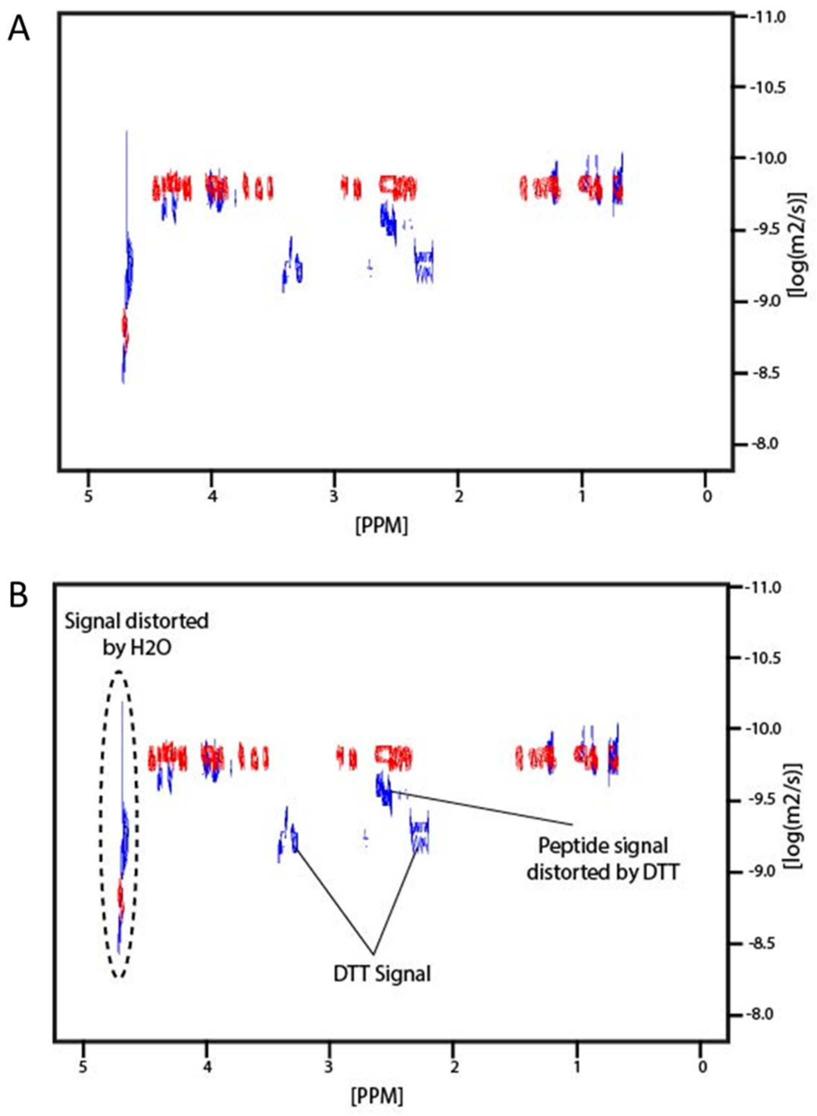

Supplementary figure 17. DOSY NMR analysis. a Overlay of DOSY NMR spectra of the oxidized **peptide 8** in red and the reduced peptide in blue. Samples were tested at a concentration of 1 mM in 0.5 X PBS with a pH = 6.0. The reduced peptide solution contained 1 mM DTT. **b** Same as in **a** but highlighting the signals distorted by water and DTT.

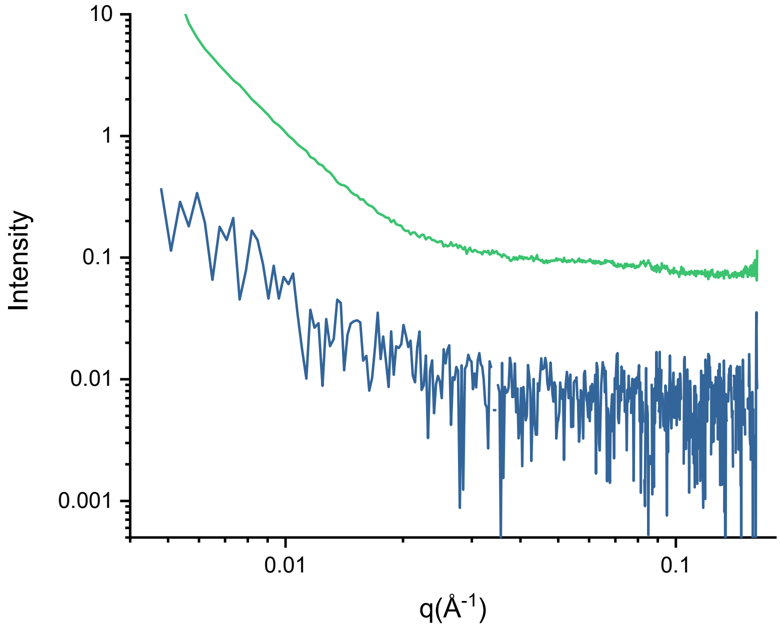

Supplementary figure 18. Small angle X-ray scattering analysis of peptide 8. Small angle X-ray scattering intensity plot of **peptide 8** (blue line) compared to the X-ray scattering of the buffer (green line), versus the scattering vector (q). **Peptide 8** is at a concentration of 1 mM in 1X PBS.

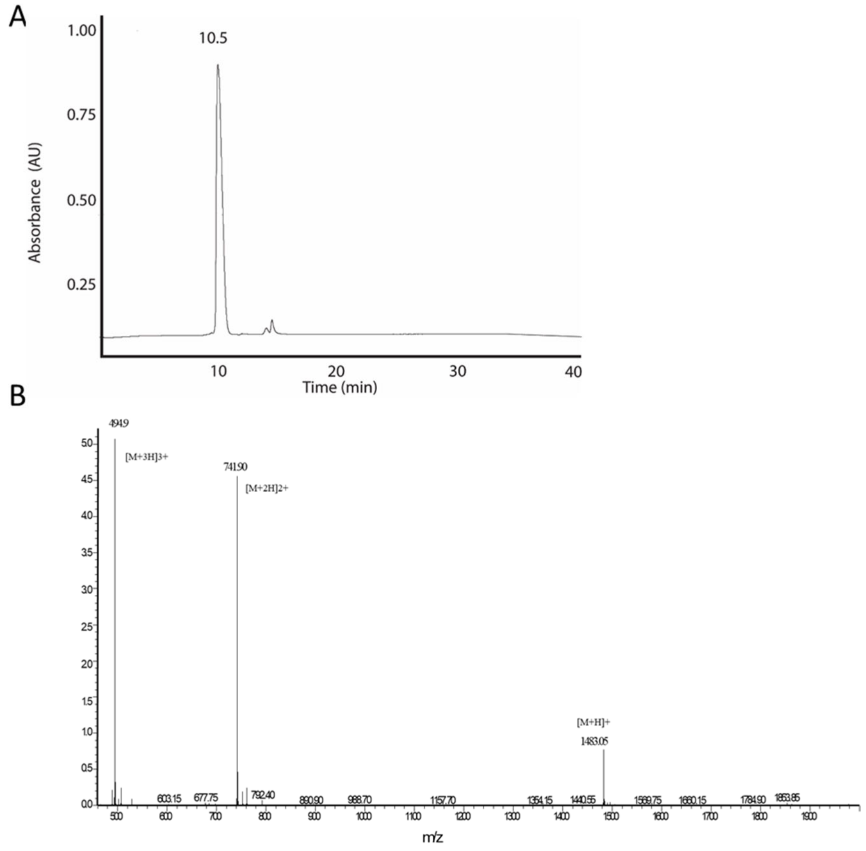

Supplementary figure 19. HPLC and LC-MS data of the Cys3-Cys13 mutant of peptide 8. a Preparative HPLC of the peptide KCSTKNCDSTAHST. **b** LC-MS data of the peptide.

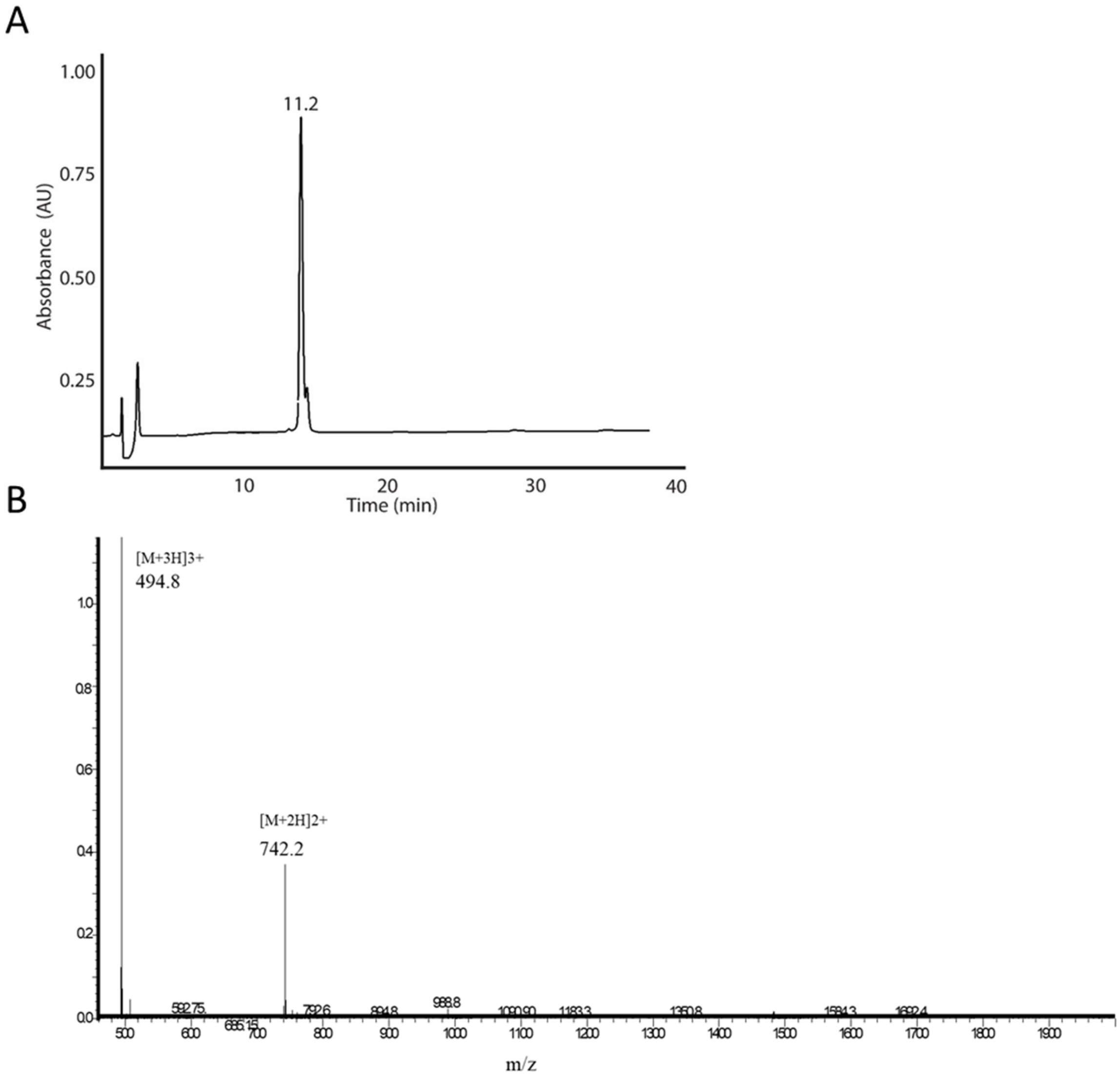

Supplementary figure 20. HPLC and LC-MS data of the Cys2-Cys7 mutant of peptide 8. a Preparative HPLC of the peptide KSCTKNSDSTAHCT. **b** LC-MS data of the peptide.

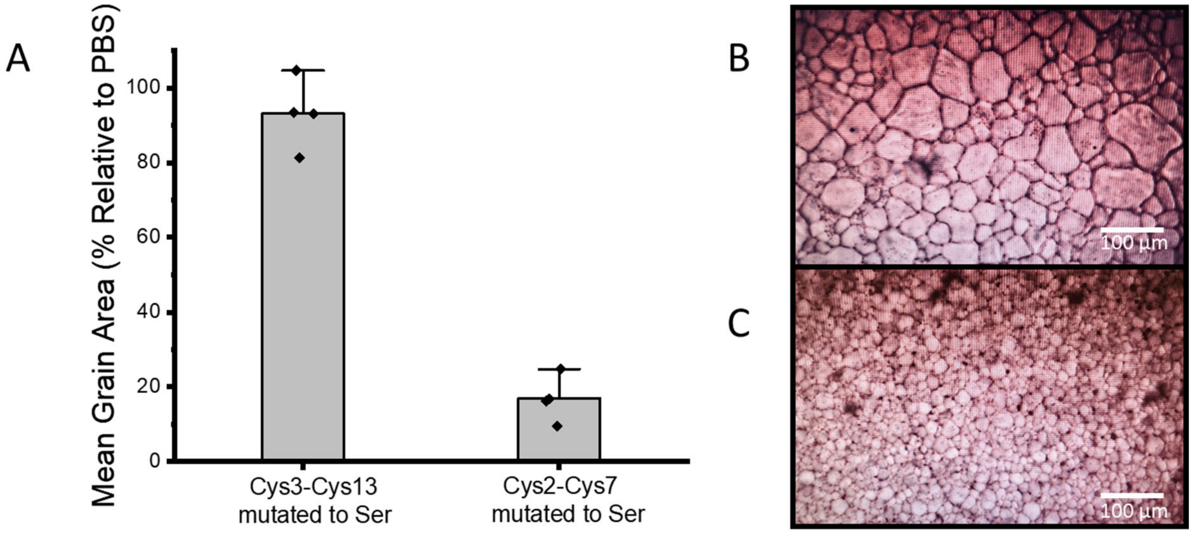

Supplementary figure 21. Ice recrystallization activities of Cys3-Cys13 and Cys2-Cys7 to Ser mutants of peptide 8. a Comparison of the mean grain area IRI activity of **peptide 8** with Cys3-Cys13 mutated to Ser and **peptide 8** with Cys-2-Cys7 mutated to Ser at 1 mg/mL (values reported are averages from three individual experiments and shown as mean data values +/- SD. Error bars represent standard deviation within the repeats). **b** Representative ice crystal grains recrystallized in the presence of Cys3-Cys13 mutated **peptide 8. c** Representative ice crystal grains recrystallized in the presence of Cys2-Cys7 mutated **peptide 8**. Experiments were performed in triplicate.

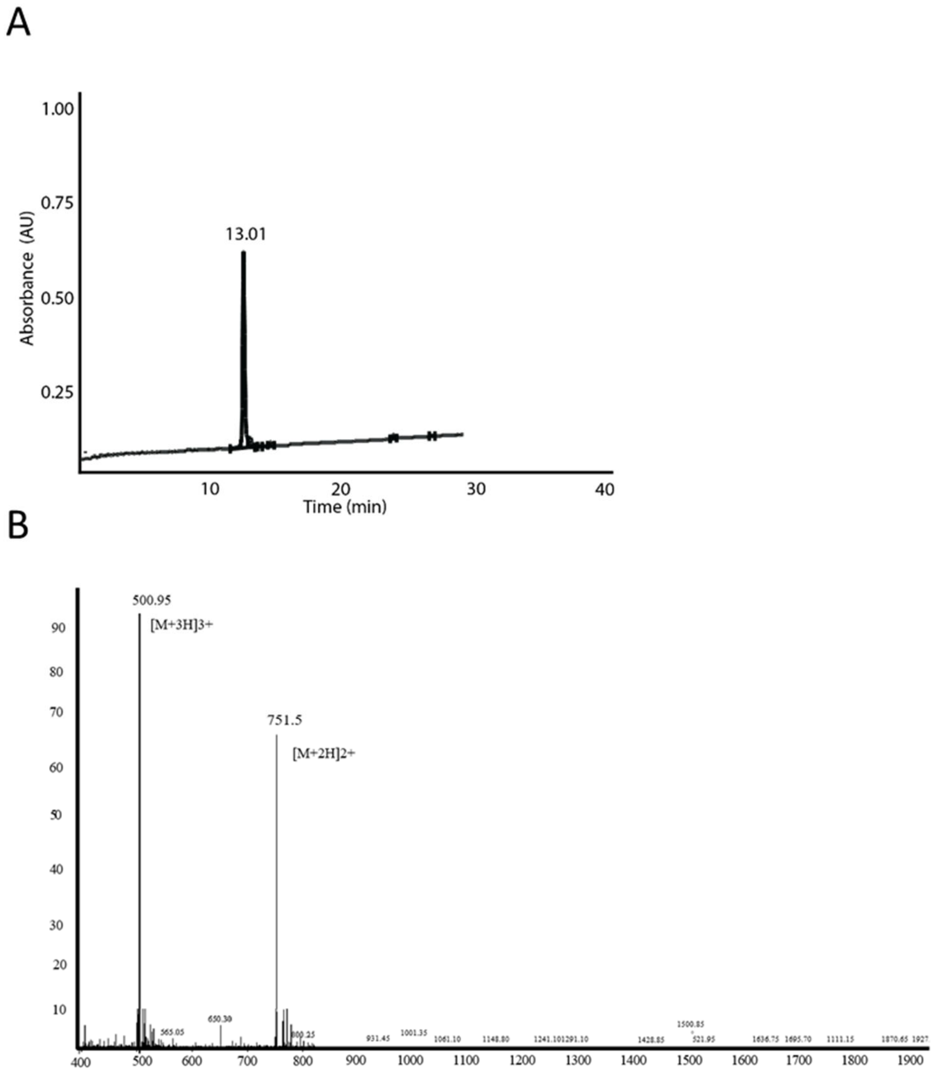

Supplementary figure 22. HPLC and LC-MS data of the Thr4 to Ser mutant of peptide 8. a Preparative HPLC of the peptide KCCSKNCDSTAHCT. **b** LC-MS data of the peptide.

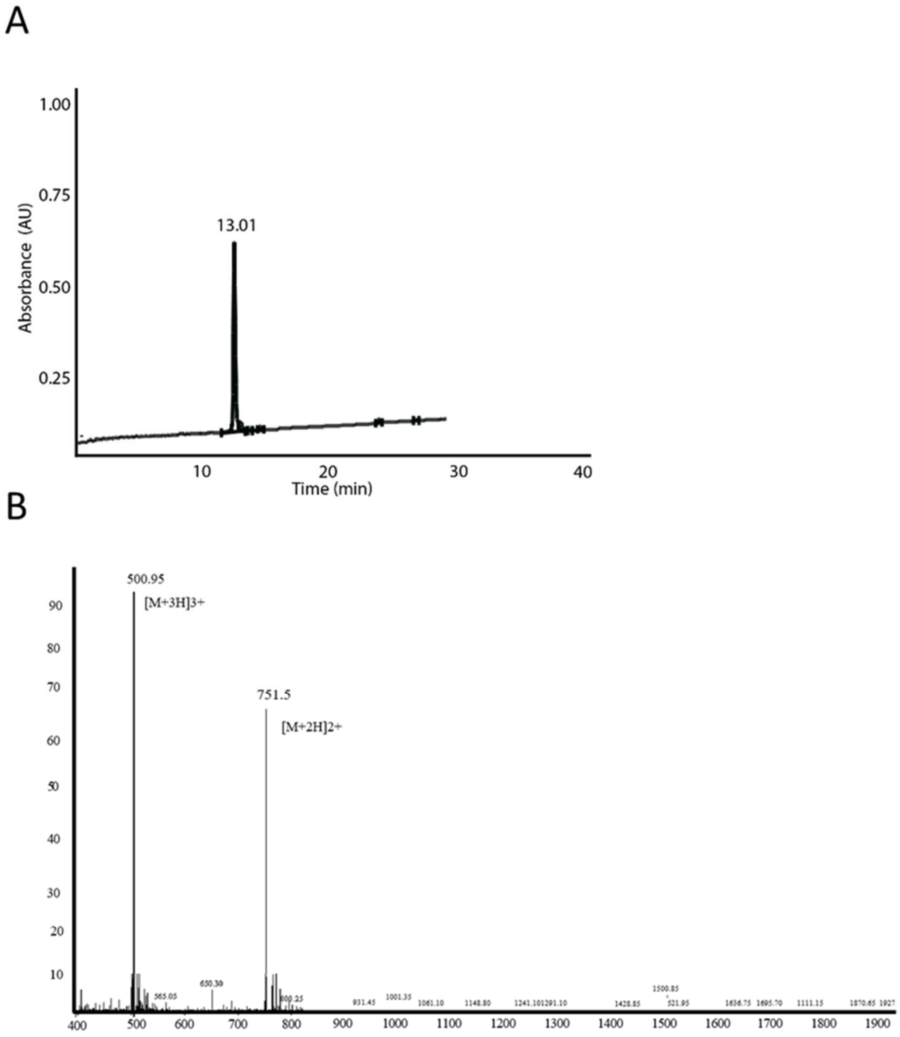

Supplementary figure 23. HPLC and LC-MS data of the Thr10 to Ser mutant of peptide 8. a Preparative HPLC of the peptide KCCTKNCDSSAHCT. **b** LC-MS data of the peptide.

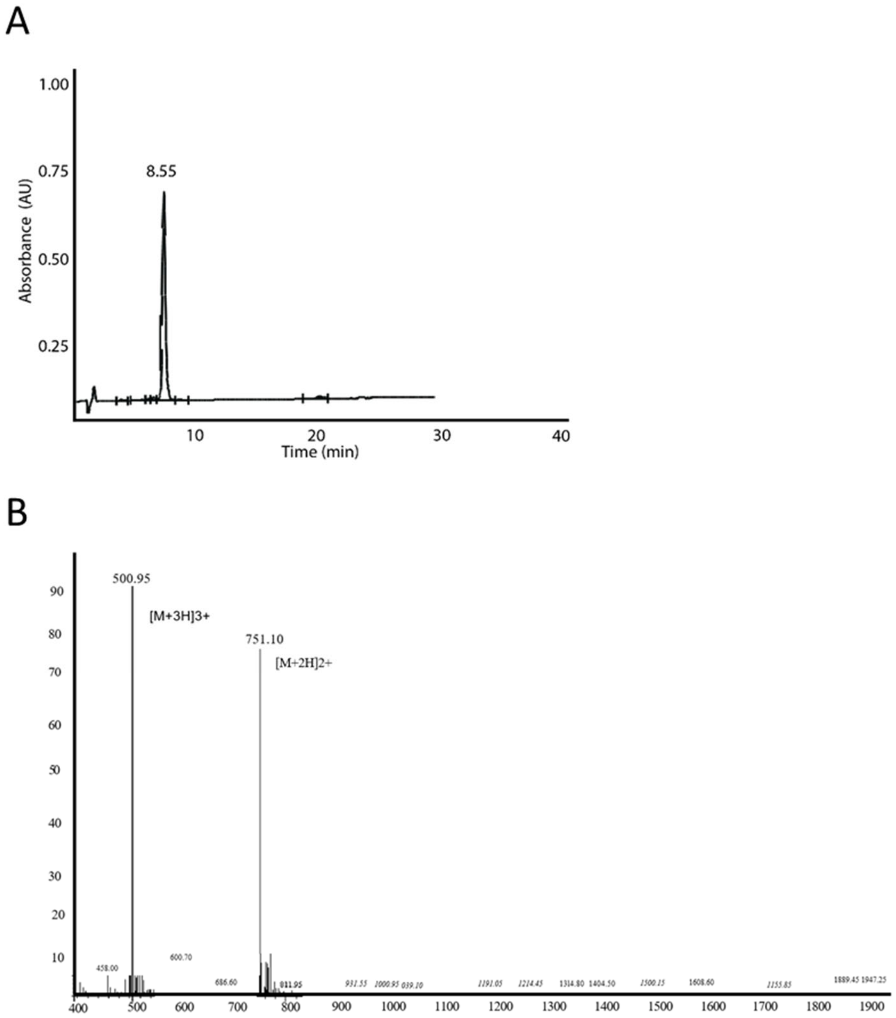

Supplementary figure 24. HPLC and LC-MS data of Thr14 to Ser mutant of peptide 8. a Preparative HPLC of the peptide KCCTKNCDSTAHCS. **b** LC-MS data of the peptide.

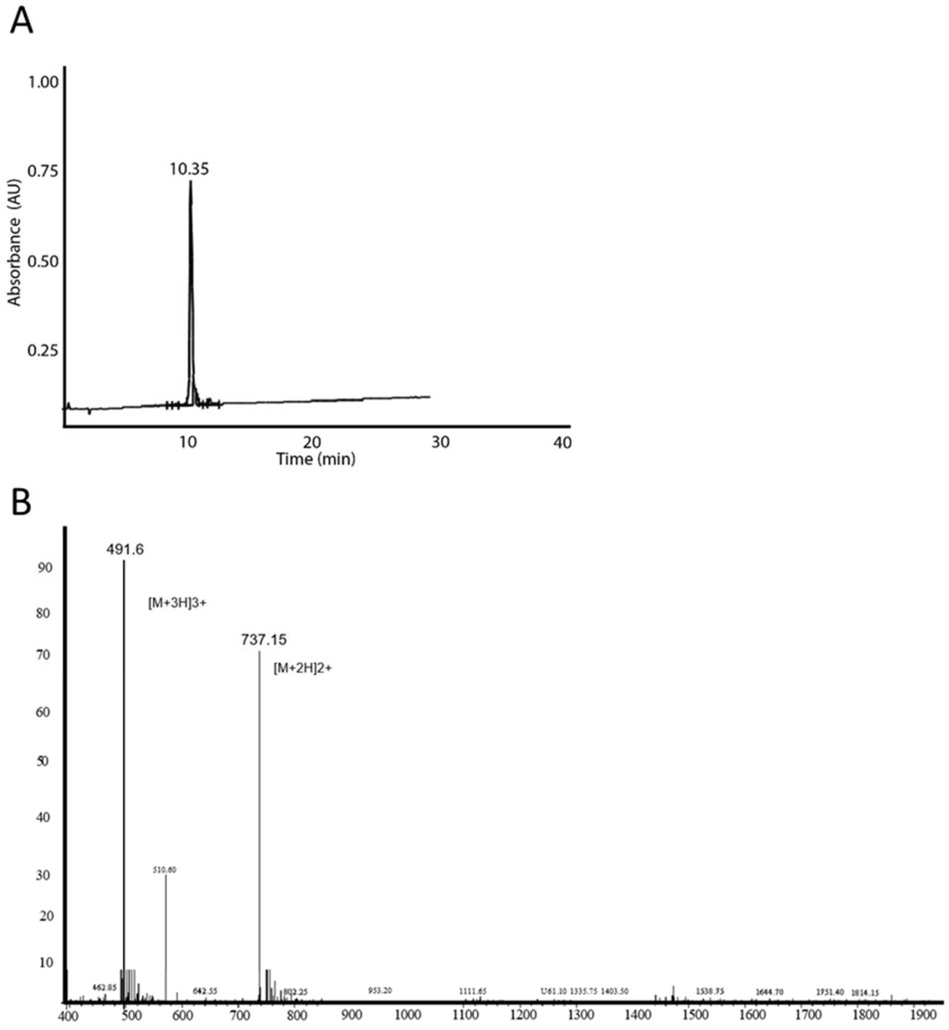

Supplementary figure 25: HPLC and LC-MS data of all Thr mutated to Ser of peptide 8. a Preparative HPLC of the peptide KCCSKNCDSSAHCS;. **b** LC-MS data of the peptide.

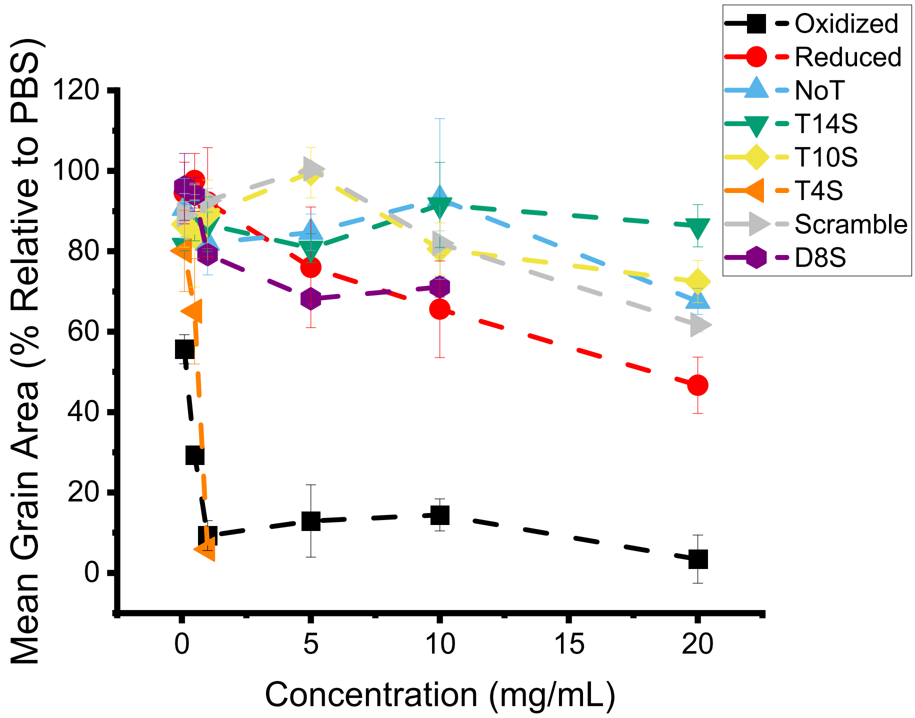

Supplementary figure 26. Dose-dependent ice-recrystallization inhibition activities. Dose-dependent response, reported as mean grain area relative to PBS, of the peptides listed in **Figure 7** (Values reported are averaged from three individual experiments and shown as mean data values +/- SD. Error bars represent standard deviation within the repeats).

A

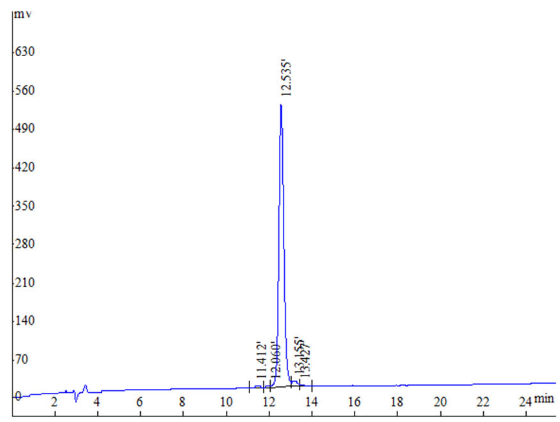

B

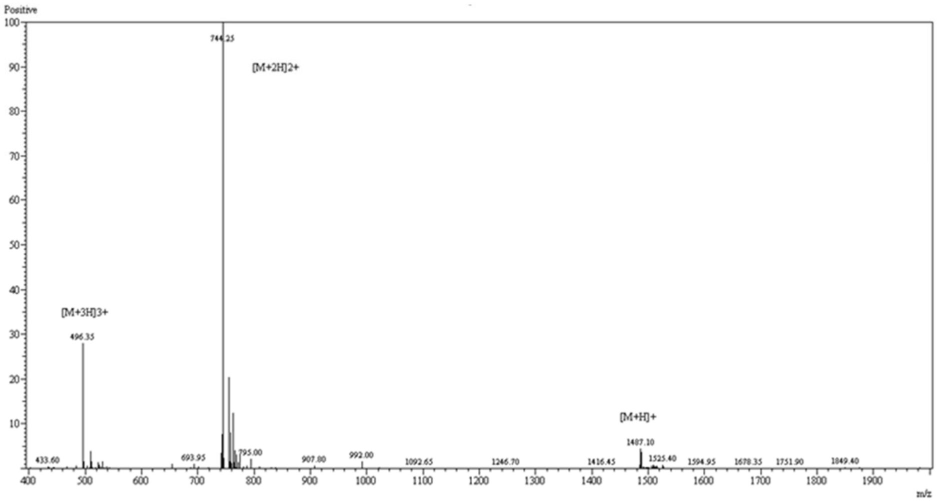

Supplementary figure 27. HPLC and LC-MS data of the Asp8 to Ser mutant of peptide 8. a Preparative HPLC of the peptide KCCTKNCSSTAHCT. **b** LC-MS data of the peptide.

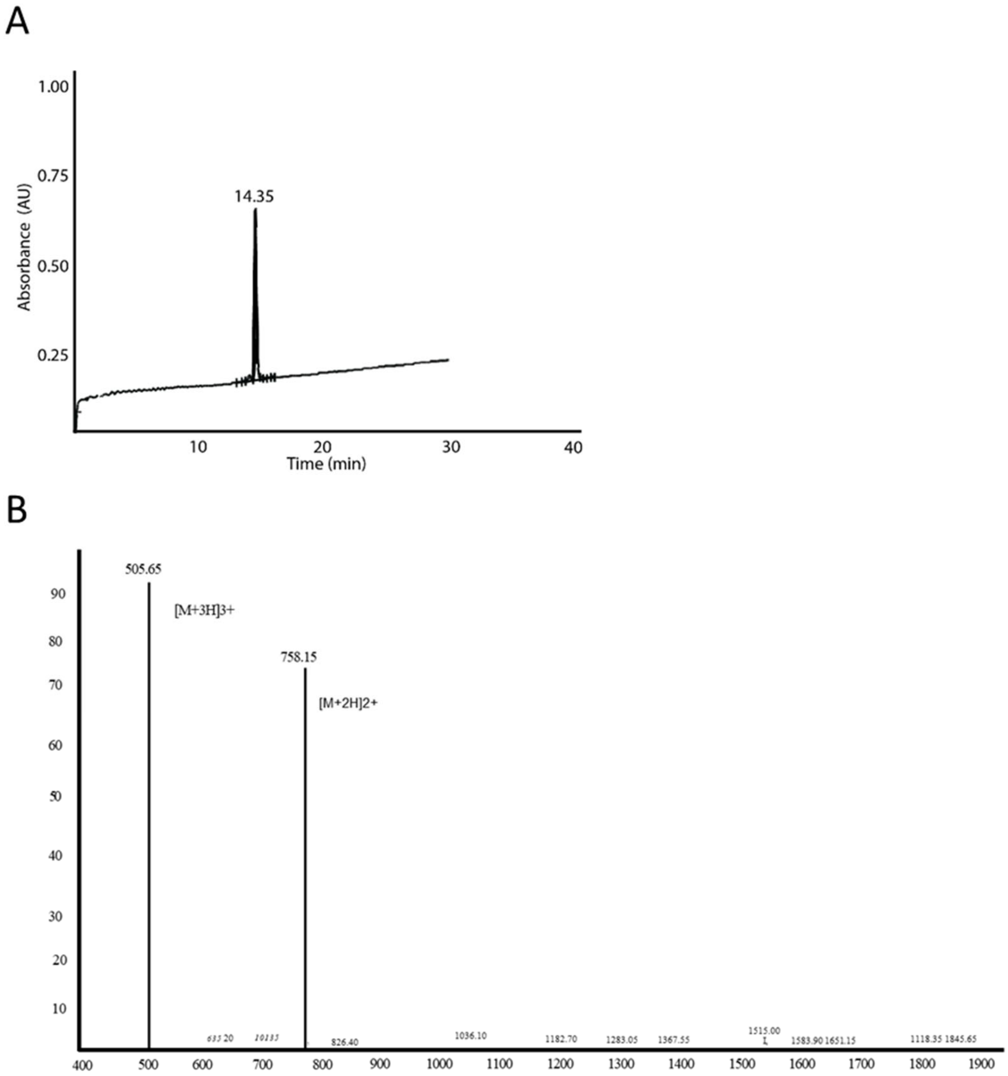

Supplementary figure 28. HPLC and LC-MS data of the scrambled sequence of peptide 8. a Preparative HPLC of the peptide TCKTDASTNCKCCH. **b** LC-MS data of the peptide.

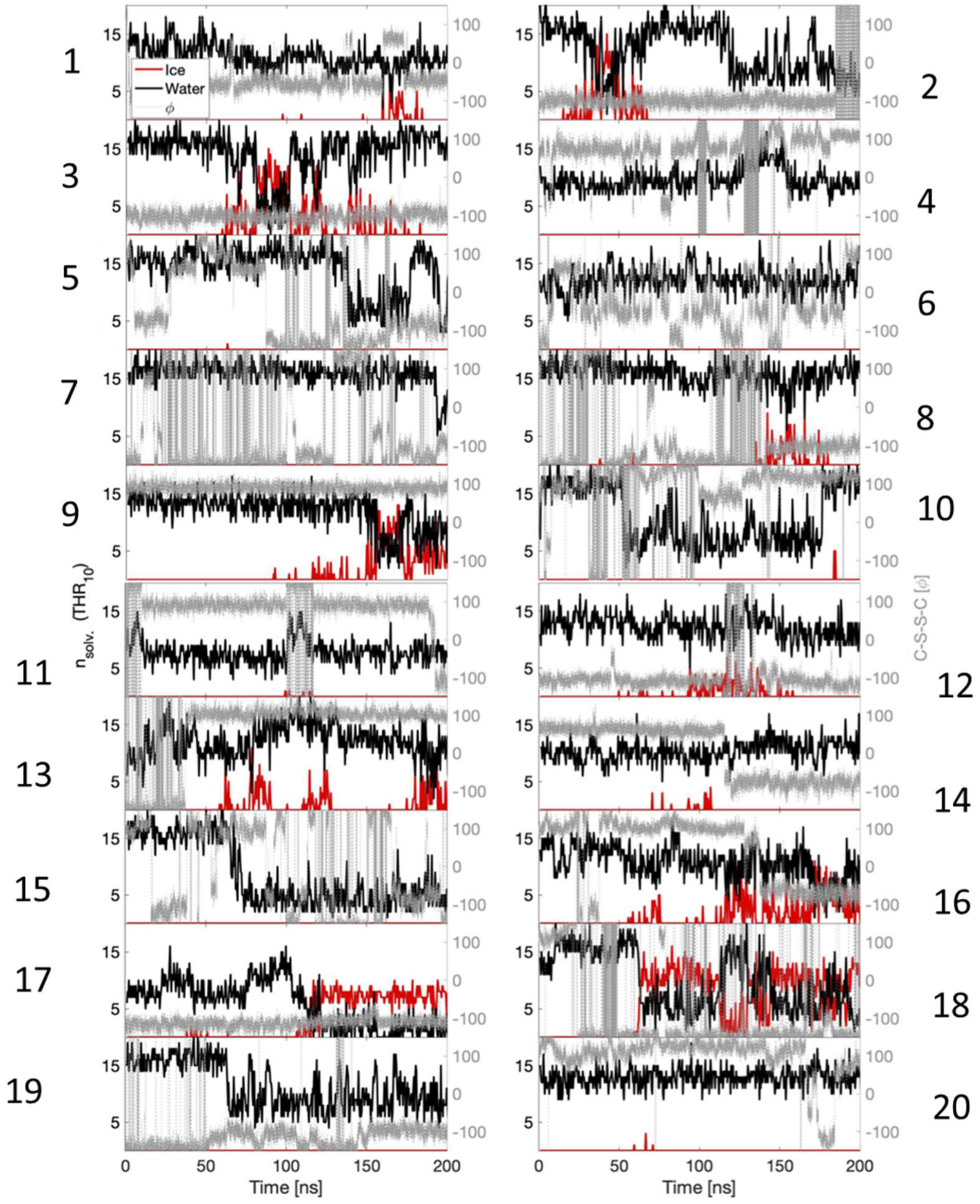

Supplementary figure 29: Plots tracking the number of ice-like and liquid waters in 20 independent molecular dynamic simulations. The red lines correspond to icy waters in the first solvation shell around the methyl group of Thr10. The black lines are the liquid waters and the grey dotted lines correspond to the C-S-S-C angle, Φ.

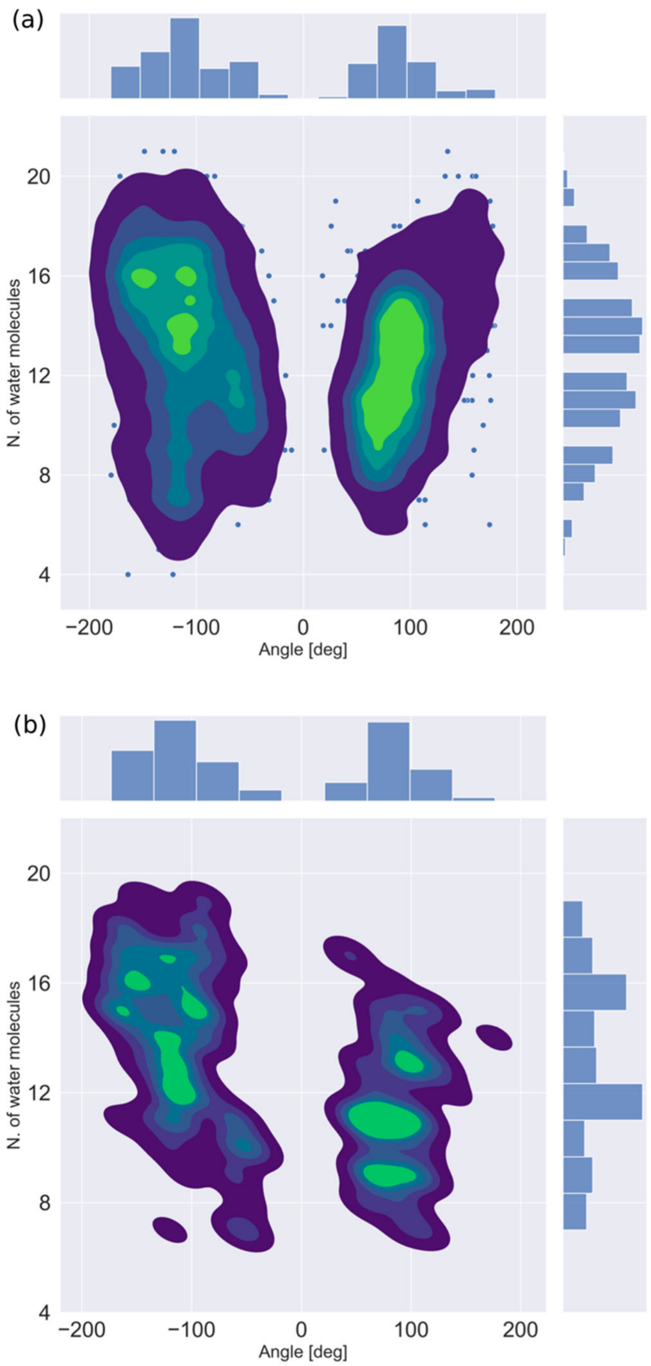

Supplementary figure 30. Contour plots generated from the 20 simulations shown in supplementary figure 29 presenting the number of water molecules within the first solvation shell of Thr10 as a function of the C-S-S-C torsional angle (φ). a Data taken along the entire MD trajectory until **peptide 8** starts interacting with ice. **b** Data taken within a restricted time section (5 ns) before the peptide binds/interacts with ice. In both instances there is a tendency for conformations characterized by negative values of the torsional angle to correspond to larger Thr10 solvation shells.

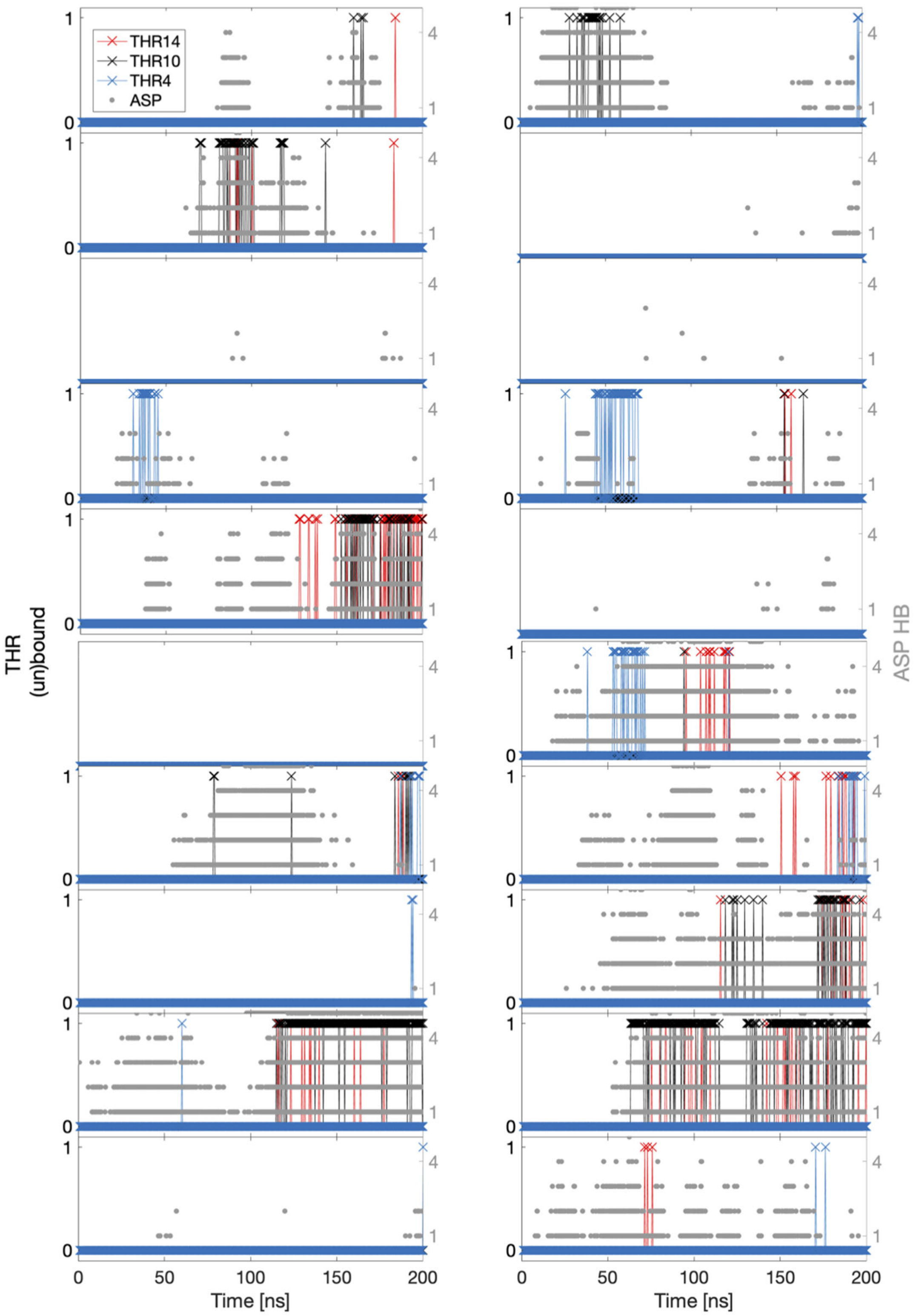

Supplementary figure 31. Plots tracking the hydrogen bonding between Asp8 and the ice throughout the simulation (right hand y-axis indicated by * in grey) along with hydrophobic bonding of the Thr groups to ice (left hand y-axis Thr14 in red, Thr 10 in black and Thr4 in blue). Hydrophobic bonding is shown as binary plots where 1 indicates the Thr residue is binding to ice and 0 indicates no binding.

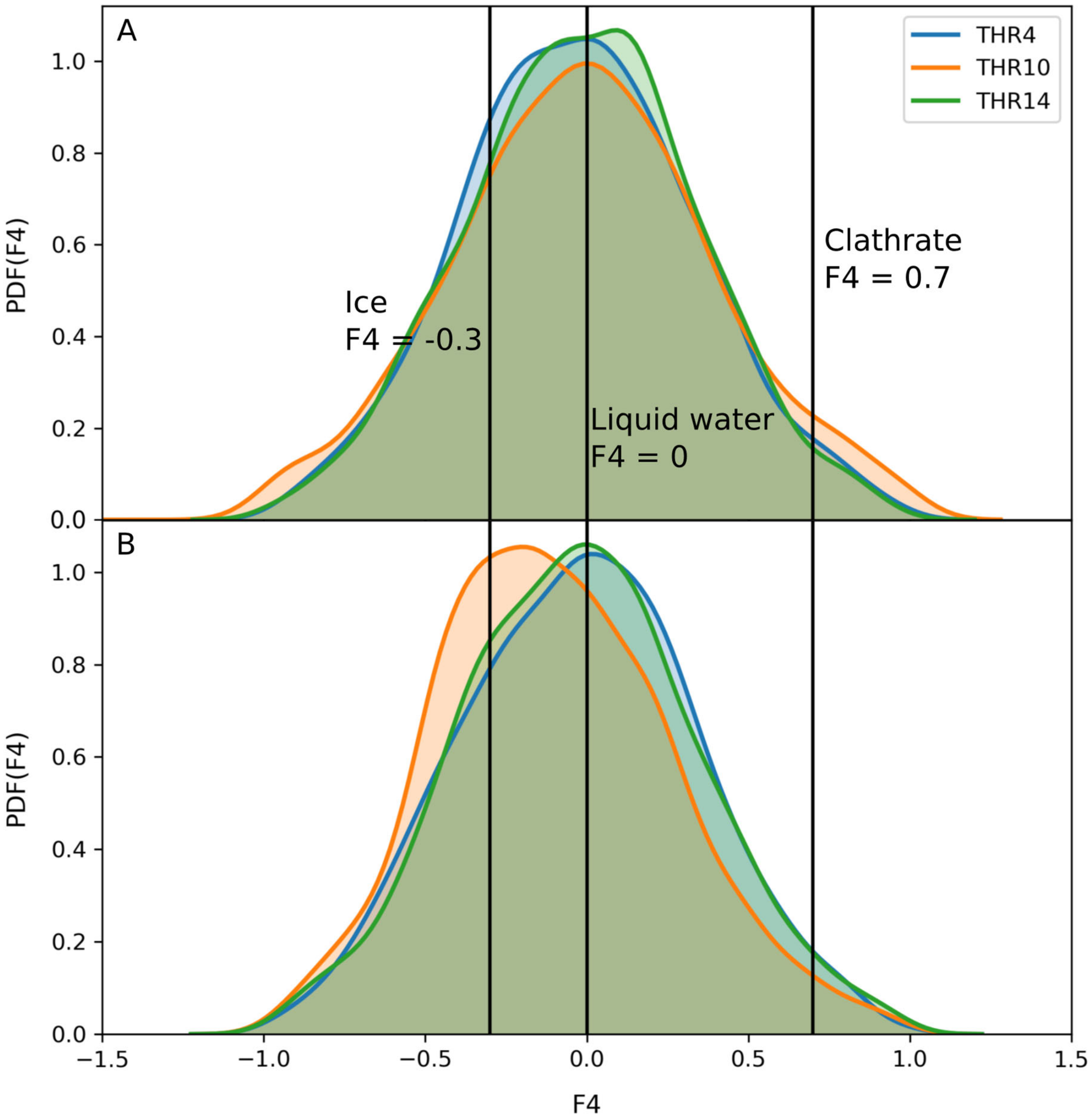

Supplementary figure 32. Probability density function (PDF) of the F_4 order parameter for the water molecules within 5 Å of each Thr residue. a Peptide in solution. **b** peptide interacting with ice. The details of the F_4 implementation are found above. Note that this result is consistent throughout all the MD trajectories that were accumulated and holds for a wide range of cut-off radii (from 3 to 15 Å) as well.

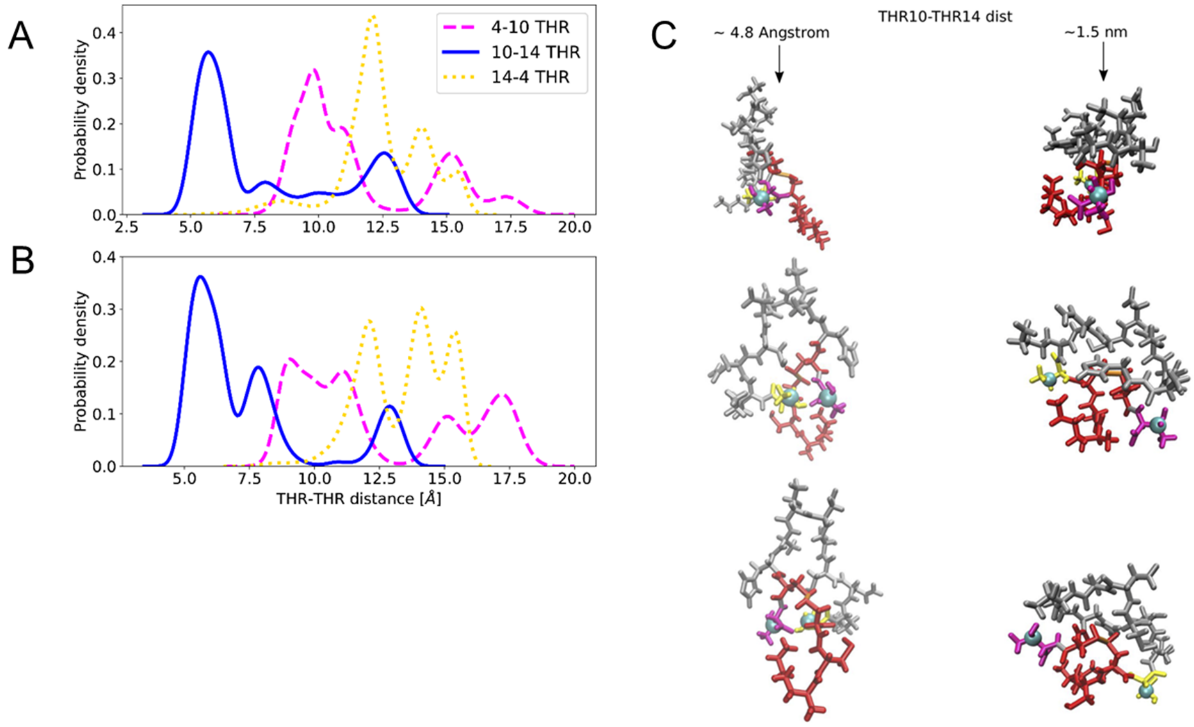

Supplementary figure 33. MD analysis of Thr – Thr distances. Probability density functions of the distances between threonine residues from the start of the simulation until the peptide binds (**a**) and during the peptide binds to ice (**b**). **c** Models showing 4.8 Å and 15 Å distance between Thr10 and Thr14 residues. Thr10 is coloured in yellow and Thr14 is coloured in magenta. Distances were calculated from the light green spheres.

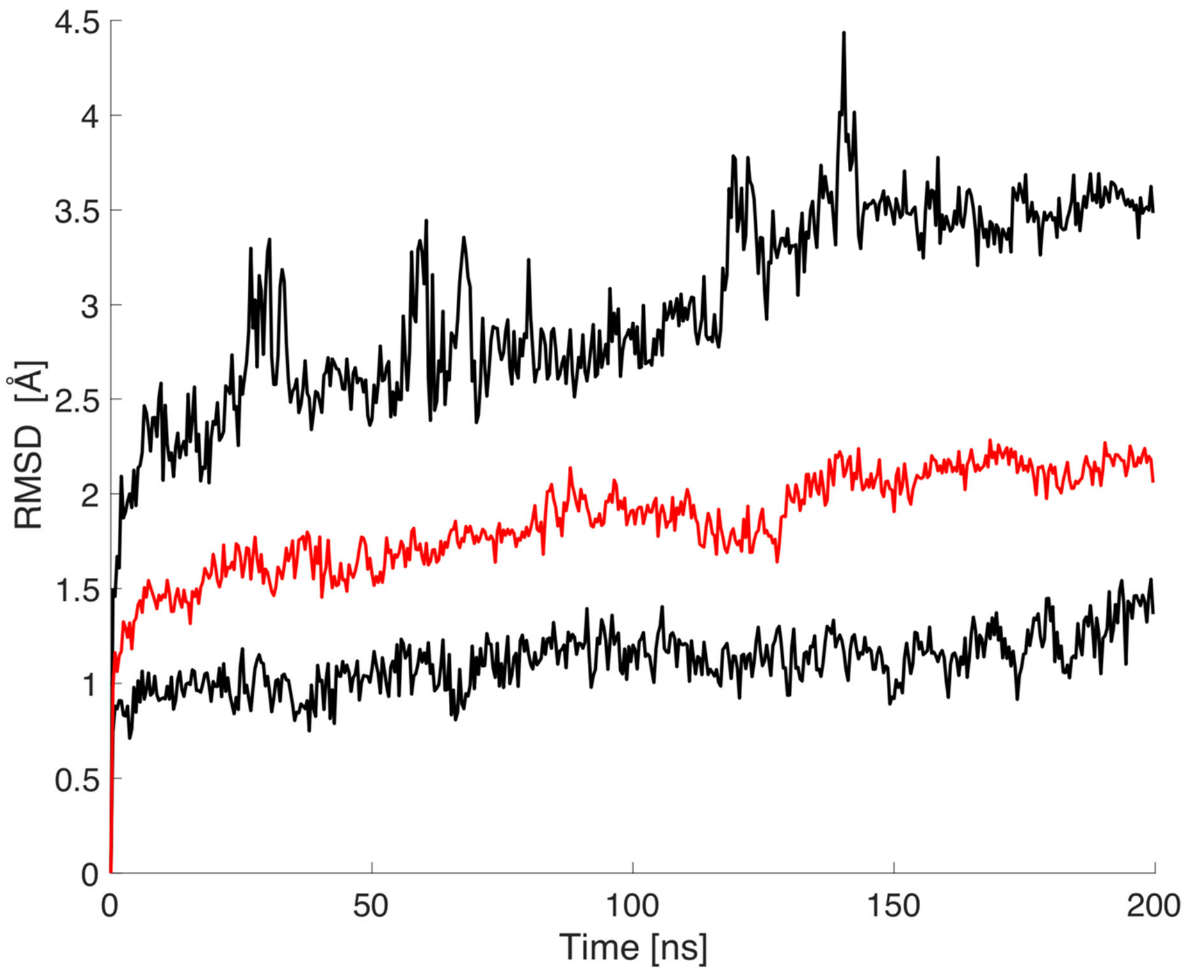

Supplementary figure 34. RMSD relative to the starting conformation. The RMSD was calculated for the peptide backbone for all 20 independent trajectories and shows the extent of structural changes as a function of time, plotted are the median (red line) min. and max. values.

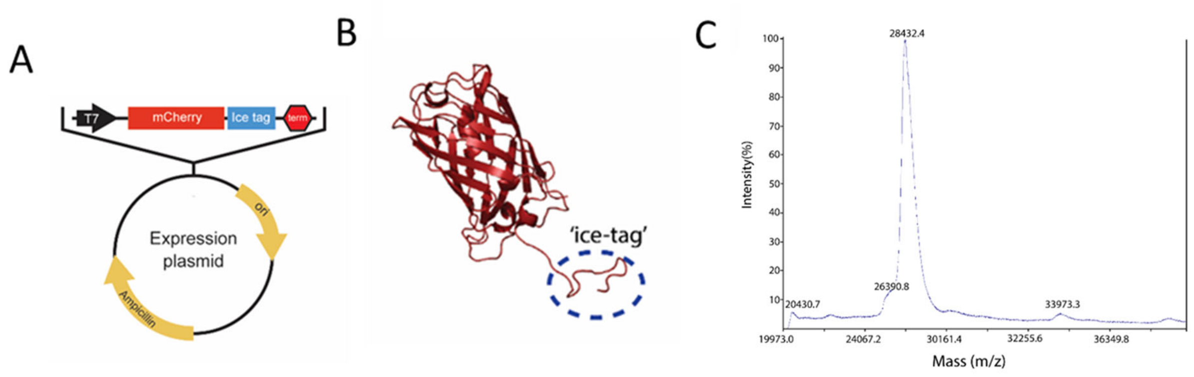

Supplementary figure 35. Structure and analysis of mCherry – ice-tag conjugate. a Plasmid map of the mCherry – ice-tag conjugate showing the incorporation of the ice-tag at the end of the mCherry sequence. **b** Cartoon representation of mCherry with C-terminal ice-tag; **c** MALDI-TOF analysis of the enriched protein after ice affinity purification.

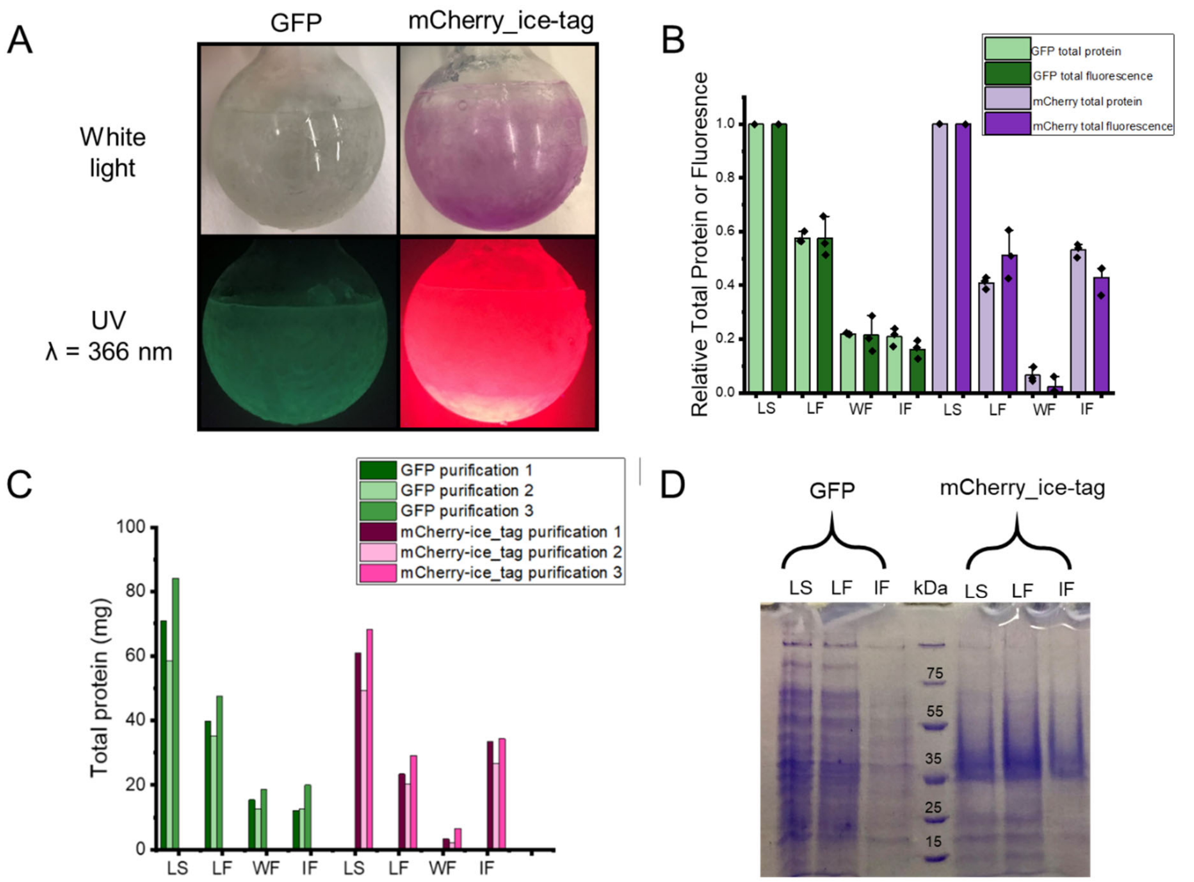

Supplementary figure 36. Rotary ice-affinity purification of GFP to mCherry_ice-tag. As a negative control for rotary ice purification *E. coli* lysate with overexpressed GFP, which shares high sequence similarity to mCherry was used. **a** Ice fraction in a round-bottom flask after incubation with GFP lysate or mCherry_ice-tag lysate imaged under both white and UV light (λ = 366 nm). **b** Plot comparing total protein in each fraction; Lysate solution (LS), Liquid Fraction (LF), Wash Fraction (WF) and Ice Fraction (IF), collected from rotary ice-affinity purification with total sample fluorescence. Shown in light green is the total amount of protein sample protein in the GFP fractions as measured by a BCA assay. In dark green is the total sample fluorescence in the GFP containing fractions as measure by a fluorimeter. In light purple is the total amount of protein in the mCherry containing fractions as measured by a BCA assay. Shown in dark purple is the total sample fluorescence for mCherry containing fractions as measured by a fluorimeter. (n=3 biologically independent samples over three independent purification experiments, data are presented as mean values +/-SD). **c** Total protein amount of rotary ice-affinity purification fractions as determined by a BCA assay. Purification 1, 2, and 3 refer to independent purification experiments of GFP lysate and mCherry-ice_tag lysate. **d** SDS-

PAGE comparison analysis of the collected GFP and mCherry fractions. Experiments were performed in triplicate.

Supplementary Tables

Supplementary table 1. Complete list of all peptide sequences identified after PCR and sequencing from four rounds of screening against ice.

Clone	Peptide Sequence	Clone	Peptide Sequence
1	SCHDCNTCGCW	45	RCDHCLCLQCS
2	SCPCEGCVNFSCF	46	RCDHCLCLQCS
3	SCPCEGCVNFSCF	47	RCDHCLCLQCS
4	SCSTCNDSVSDCCL	48	RCDHCLCLQCS
5	SCSTCNDSVSDCCL	49	RCDHCLCLQCS
6	SCSTCNDSVSDCCL	50	RCDHCLCLQCS
7	SCSTCNDSVSDCCL	51	RCDHCLCLQCS
8	SCSTCNDSVSDCCL	52	RCDHCLCLQCS
9	SCSTCNDSVSDCCL	53	RCPFPCTPQRCCY
10	TCSRTHQCTRPCCP	54	RSCWRCSSAPFCCA
11	TCSRTHQCTRPCCP	55	KCCTKNCDSTAHCT
12	TCSRTHQCTRPCCP	56	KCCTKNCDSTAHCT
13	TCSRTHQCTRPCCP	57	KCCTKNCDSTAHCT
14	TCSRTHQCTRPCCP	58	KCCTKNCDSTAHCT
15	TCCAFCVSCI	59	KCCTKNCDSTAHCT
16	TCCAFCVSCI	60	KCCTKNCDSTAHCT
17	TCCAFCVSCI	61	KCCTKNCDSTAHCT
18	TCCAFCVSCI	62	KCCTKNCDSTAHCT
19	TCCAFCVSCI	63	KCCTKNCDSTAHCT
20	TCCAFCVSCI	64	KCCTESPLCCT
21	TCCAFCVSCI	65	KCCTESPLCCT
22	GCCCGSSVCH	66	KCCTESPLCCT
23	GCVSCCQGTCF	67	KCCNVINCCK
24	GCVSCCQGTCF	68	KCCNVINCCK
25	GCVSCCQGTCF	69	KCCNVINCCK
26	GCVSCCQGTCF	70	KCCNVINCCK
27	GCVSCCQGTCF	71	KCCNVINCCK
28	ACSDRFRNCPADEALCG	72	KCCNVINCCK
29	ACSDRFRNCPADEALCG	73	KCCNVINCCK
30	ACSDRFRNCPADEALCG	74	KCCNVINCCK
31	ACSDRFRNCPADEALCG	75	KCCNVINCCK
32	ACSDRFRNCPADEALCG	76	KCCNVINCCK
33	ACSDRFRNCPADEALCG	77	KCCNGICCE
34	ACSDRFRNCPADEALCG	78	VCCLHSSEICDCS
35	ACSDRFRNCPADEALCG	79	DCCWQGWCICN
36	ACSDRFRNCPADEALCG	80	MCANCSEDPCCS
37	ACSDRFRNCPADEALCG	81	NCHPCNNCTNCK
38	ACSDRFRNCPADEALCG	82	QCRTQPSCPNCKCK
39	ACSDRFRNCPADEALCG	83	QCMRQPCENCCR
40	ACSDRFRNCPADEALCG	84	QCMRQPCENCCR
41	ACSDRFRNCPADEALCG	85	QCMRQPCENCCR
42	ACSDRFRNCPADEALCG	86	QCMRQPCENCCR
43	ACSDRFRNCPADEALCG	87	QCMRQPCENCCR
44	ACSDRFRNCPADEALCG	88	QCMRQPCENCCR

Supplementary table 2. Backbone chemical shifts of peptide 8 as determined by NMR (ppm).

Residue	aa	HN	N	CA	CB	HA
1	K	7.636	119.43	55.66	29.26	4.08
2	C	9.011	122.59	58.75	28.13	4.6
3	C	8.65	117.78	54.9	38.84	4.73
4	T	8.451	116.46	62.1	69.65	4.35
5	K	8.524	123.97	56.51	29.23	4.33
6	N	8.704	120.49	53.26	38.75	4.71
7	C	8.549	119.84	59.11	28.12	4.55
8	D	8.951	123.45	58.61	42	4.64
9	S	8.463	117.61	58.79	63.73	4.5
10	T	8.299	115.56	62.11	69.63	4.34
11	A	8.261	125.93	52.85	19.26	4.27
12	H	8.639	121.81	53.27	28.95	4.77
13	C	8.587	121.05	58.75	42.07	4.63
14	T	8.218	118.05	62.11	70.14	4.31

Supplementary table 3: Phage sequencing primers

Primer (5' to 3')	Sequence																					
Forward	C	T	G	G	C	G	C	T	G	A	A	A	C	T	G	T	T	G	A	A	A	G	
Reverse	G	C	T	T	C	A	T	C	T	G	C	C	G	G	A	C	A	A	T	T	A	C	G

Supplementary Methods

Materials

Peptides. Peptide 1 and **peptides 5-9** were synthesized in house by solid phase peptide synthesis (SPPS). **Peptides 2-4**, **peptide 8** (in addition to the **peptide 8** synthesized in house) and all **peptide 8** mutants (Cys2-Cys7, Cys3-Cys13, Thr4-Ser, Thr10-Ser, Thr14-Ser and the scrambled peptide) were synthesized by GLBiochem (Shanghai, Ltd Address: 519 Ziyue Road, Minhang Shanghai 200241 China). These peptides were subsequently re-injected onto an HPLC and their mass was confirmed by LC-MS.

Other materials and reagents. Dried phosphate buffered saline (PBS) tablets were purchased from VWR® (PanReac, AppliChem) and dissolved in MilliQ water according to the directions resulting in the following final concentrations: 157 mM Na^+, 140 mM Cl^-, 4.45 mM K^+, 10.1 mM HPO_4^{2-}, 1.76 mM $H_2PO_4^-$ at a pH = 7.6. The resulting solution was termed 1X PBS. Additionally, this solution was further diluted 1:2 with MilliQ water resulting in with reduced salt concentrations and was termed 0.5X PBS solution.

Gene construct. mCherry-Ice_tag gene was purchased from GeneArt® (ThermoFisher Scientific).

Phage displayed peptide libraries. Peptide libraries XD9 to XD14 were constructed as previously described[1]. The libraries had the peptide format of $XCX_mCX_nCX_oCX$ ($m + n + o$ = 3, 4, 5, 6, 7 or 8; where X indicates random amino acids, C indicates cysteine). The libraries are constructed from a filamentous phage fd, which displays peptides on the N terminus of the pIII coat protein at the end of the viral particle[2]. In total, five peptides are displayed per viral particle.

***Escherichia coli* TG1:** All phage display experiments utilized *E. coli* TG1 cells for phage amplification and titer.

Procedures

Ice shell formation. The ice shell used in the screening and purification experiments were formed as previously described in Marshall *et al.*[3] Briefly, A 250 mL round-bottom flask was filled with 100 mL of distilled and deionized water. The flask was rotated in an ethanol dry-ice bath (approximately -78 °C) for 45 seconds. The remaining liquid was poured into a graduated cylinder to determine the amount of ice formed on the inside of the flask, on average approximately 10 mL of water would freeze. Next, the flask was

rotated in the ethanol-dry ice bath for an additional 45 seconds. The round bottom flask with the newly formed ice shell was transferred to the cooling bath or stored at -20 °C for future use.

Phage selection. Phage libraries XD9 to XD14 were produced on a 0.5 L scale and purified as described by R. Rebollo *et al.*[4] Briefly, for each library XD9 to XD14, a 1 mL *E. coli* TG1 glycerol stock was inoculated 0.5 L 2×YT media (containing 10 μg/mL tetracycline) and grown overnight (16 hours) at 30 °C with 200 rpm shaking. Next the *E. coli* TG1 cells were removed by centrifugation at 4640 ×g for 20 min at 4 °C, while the phages remained in the supernatant. After that, the supernatant was decanted in a new flask and mixed with 125 mL of precipitation buffer (20% PEG 6000, 2.5 M NaCl). The mixture was incubated on ice for 30 min. Then, the precipitated phage mixture was centrifuged at 4640 ×g for 45 min at 4 °C. After the centrifugation, the supernatant was discarded and the phage pellet of each library was dissolved in 15 mL of 1 X PBS. A 100 μL aliquot of each phage library (in total 600 μL for XD9 to XD14) was added to 50 mL of distilled and deionized water. This solution was cooled to and held at 0 °C for 30 min. Next, this solution was added to a 100 mL round-bottom flask with a pre-formed ice shell (approx. 10 mL) in the interior of the flask. The solution was rotated in ethylene glycol chilled to -0.5 °C for approximately 2 min. As phages have limited cryotolerance, a brief two-minute incubation phase was selected to allow for binding to ice but limit the amount of ice overgrowth to preserve phage function. The remaining liquid was poured from the round-bottom flask. Next, the ice was washed with five 50 mL aliquots of wash buffer (three aliquots of 1X PBS, and two aliquots of 1 X PBS + 0.2 % (*v*/*v*) Tween-20) pre-cooled to 0 °C. Phages were eluted by melting the ice at room temperature for 30 min. To amplify phages for additional rounds of screening and sequencing, eluted phages (approx. 10 mL) were incubated with 30 mL *E. coli* TG1 cells (OD_{600}=0.4) for 90 min at 37 °C without shaking. Next, the solution of phage-infected bacteria was pelleted by centrifugation. The supernatant was discarded and the pellet was resuspended in 1 mL of fresh 2 × YT media. A 20 μL aliquot of the resuspension was removed and serially diluted (10^1 to 10^8). The dilutions were plated onto 2 × YT / tetracycline plates. Phage DNA contains a gene for tetracycline resistance, which post infection, allows the bacteria to grow in the presence of the antibiotic. The resulting colonies were counted (CFU) to quantify the number of phages that bound to the ice. The remaining 980 μL solution phage-infected *E. coli* TG1 cells were plated on large

(100 mm in diameter) 2 × YT / tetracycline (10 μg mL^{-1}) plates, to select for phage-infected bacteria. The following day, phage-infected bacteria were collected from the large agar plates by scrapping into a solution of 2×YT/tetracycline + 10% glycerol. This solution was aliquoted into 1 mL cryovials and snap frozen for storage at -80 °C. Subsequently, a single 1 mL glycerol stock was used to inoculate a 0.5 L culture of 2×YT/tetracycline and grown to an O.D = 0.4. Phages were purified from this solution by precipitation as described above and used in subsequent rounds of selection. Four additional rounds of selection were performed using the same procedure. After four rounds of screening, single colonies were picked from the 2×YT/tetracycline plates, and the DNA of clones was amplified by PCR. The primers used for amplification are listed in supplementary table 3. Subsequently, the amplified DNA was and sequenced by Macrogen (Amsterdam, Netherlands) as previously described[1].

Peptide synthesis, cleavage and deprotection. Peptides were synthesized on an Advanced ChemTech 348Ω parallel peptide synthesizer (AAPPTec) by standard Fmoc solid-phase chemistry on Rink Amide resin (0.26 mmol g^{-1} resin, 0.03 mmol scale), using DMF (99.5% pure) as a solvent. The coupling was carried out twice for each natural amino acid (4 equiv.) using HBTU (4 equiv.), HOBt (4 equiv.) and DIPEA (6 equiv.). Fmoc groups were removed using a 20% v/v solution of piperidine in DMF. Washing steps were performed with DMF. Peptides were deprotected and cleaved from the resin under reducing conditions (90% TFA, 2.5% H_2O, 2.5% thioanisole, 2.5% phenol, 2.5% EDT) for 4 h with shaking at RT. The resin was removed by vacuum filtration, and the peptides were precipitated in 50 mL of cold diethyl ether and incubation for 30 min at −20 °C and pelleted by centrifugation. The precipitated peptides were washed twice with cold diethyl ether (30 and 20 mL, respectively). Precipitated peptides were dissolved in an equal parts mixture of H_2O and TFA prior to purification.

Peptide purification. Peptides were purified by semi-preparative reverse-phase HPLC (PrepLC 4000-Waters system, Vydac C18 TP1022, 250 × 22 mm, 10 μm) using a flow rate of 20 ml min^{-1} and a linear gradient of 5-90% v/v solvent B in 30 min (A: 99.9% v/v H_2O and 0.1% v/v TFA; B: 99.9% v/v MeCN and 0.1% v/v TFA). The mass of purified peptides was determined by electrospray ionization mass spectrometry (ESI-MS) in positive ion mode on a single quadrupole liquid chromatograph mass spectrometer (LCMS-2020,

Shimadzu). Fractions containing the desired peptide were lyophilized. Prior to the activity assay, peptides were dissolved in 1X PBS and left on the bench-top at room temperature for >3 hours to allow for oxidation.

Splat Ice Recrystallization Inhibition Assays. The splat ice recrystallization inhibition was preformed as detailed previously detailed.[5] Briefly, a 10 μL droplet of peptide solution in 1 X PBS solution was dropped from 2 m onto a glass microscope coverslip, which is atop of an aluminum plate cooled to −78 °C using dry ice. The droplet freezes instantly upon impacting the coverslip, spreading out and forming a thin wafer of ice. This wafer was then transferred to a liquid nitrogen cooled cryostage and held at − 8 °C for 30 min. After annealing three photographs are taken of the wafer. The number of crystals in the image are counted, with ImageJ software. The area of the field of view is divided by the number of crystals to give the average crystal size per wafer, reported as a percent of area compared to PBS control.

Sucrose sandwich IRI assays. Sucrose sandwich IRI assays were performed as described by Stevens *et al.*[6] Briefly, peptide samples in 1 X PBS (pH=7.6) containing 40% sucrose were sandwiched between two coverslips, and the edges were sealed with grease. Samples were cooled to, and held at, −50 °C for 2 min on a Linkam stage temperature-controlled cold stage (Linkam Scientific Instruments). The temperature was then elevated to −8 °C and held for 2 h and subsequently imaged.

NMR. Lyophilized peptide for resonance assignment experiments was dissolved to a final concentration 6 mM in 0.5 X PBS buffer pH 6 with 10% D_2O (deuterium oxide) added. For DOSY (diffusion ordered spectroscopy) experiments, the peptide was dissolved to 1 mM in 20 mM sodium phosphate buffer pH 6 prepared in 100% D_2O. All experiments were carried out in a Bruker 500 MHz spectrometer equipped with a 5 mm ^{1}H,^{13}C,^{15}N BBI probe and an Avance Neo console. Resonance assignments were achieved by analyzing ^{1}H-^{1}H TOCSY (total correlation spectroscopy) (60 ms spin lock), ^{1}H-^{1}H NOESY (nuclear overhauser effect spectroscopy) (120 and 200 ms mixing time), natural abundance ^{1}H-^{13}C HSQC (heteronuclear single quantum coherence) and natural abundance ^{1}H-^{15}N HSQC, all acquired at 280 K using the standard Bruker pulse sequences dipsi2esgpph, noesyesgpph, hsqcetgp and hsqcetfpf3gpsi, respectively. These spectra were recorded on standard

spectral windows for diamagnetic protein shifts, with 64 indirect dimension increments and 128 scans for the natural abundance ^{1}H -^{15}N HSQC, 256 increments and 32 scans for the natural abundance ^{1}H -^{13}C HSQC, and 256 increments and 8 scans for ^{1}H-^{1}H correlation spectra. These spectra were acquired and processed with TopSpin 4.0 and manually analyzed for resonance assignment using CARA 1.9.1. The final assignment included backbone and CB chemical shifts for all residues, and 95 % completion for sidechain nuclei. These chemical shifts were used for structure calculation on the chemical shift-Rosetta server available at BioMagResBank. DOSY experiments were acquired (also at 280 K) using Bruker's ledbpgp2s sequence with a diffusion gradient of 120 ms, a diffusion time of 15000 μs and a linear gradient ramp, and processed with Topspin 4's built-in module.

Circular Dichroism (CD) spectroscopy. CD was measured using a Chirascan CD Spectrometer (Applied Photophysics, Leatherhead Surrey, UK) at room temperature. Ten scans between 195-260 nm were collected of oxidized peptide 8 at 1 mM concentration in 1X PBS. Ten scans between 200-260 nm were collected for the reduced peptide 8 at 1 mM concentration 1X PBS+10 mM DTT, as the DTT gave a signal below 200 nm. In each case, the ten scans were averaged, and buffer-reference subtracted.

Bicinchoninic acid (BCA) protein assay. A Thermo Scientific^{tm} Pierce^{tm} BCA protein assay kit (#23225) was used to quantify the total amount of protein in the fractions collected during rotary ice-affinity purification. The assay was completed as per the included instructions. A standard curve for estimating total protein concentration was developed based on bovine serum album (BSA) with a working range of 20-2000 μg/mL. All samples were prepared by mixing 20 μL of the standard or unknown sample with 200 μL of the working reagent in a 96-well plate and incubating at 37 °C for 30 min (Greiner 96-well Flat bottom Transparent Polystyrene). After incubation all samples were measured in duplicate at 562 nm by a Tecan plate reader (Infinite 200 Pro multimode reader). Total protein was calculated using the BSA standard curve and converted into milligrams via multiplying by the sample volume.

Quantification of sample fluorescence. The fluorescence of each fraction collected during the rotary ice-affinity purification was measured using a Tecan plate reader

(Infinite 200 Pro multimode reader). Briefly, 200 µL of each sample was aliquoted into 96-well plates in duplicate (Greiner 96-well Black Flat Bottom Transparent Polystyrene). The fluorescence of GFP containing fractions was measure by excitation at 488 nm and emission was recorded at 509 nm. The fluorescence of mCherry containing fractions was measured by excitation at 540 nm and emission at 605 nm. Total sample fluorescence was calculated by multiplying by sample volume.

Molecular dynamics simulations. Simulations were preformed using the all-atomistic CHARMM36 forcefield,[7] along with the TIP4P/Ice water model[8] and using the MD package GROMACS 5.1.3.[9] The analysis of the results involved with the computational aspects of this work has been performed using PLUMED (version 2.4.2), MDAnalysis (version 1.0.0) and MATLAB (version 9.9.0.1495850, R2020b). There is a large body of evidence indicating that the CHARMM36 - TIP4P/Ice combination of force fields is especially well suited to perform MD simulations of biomolecules in supercooled water (and in contact with ice).[10–13] We have ran 20 independent simulations, each one 200 ns long for **peptide 8**. To begin with, the geometry of a **peptide 8** was optimised using a steepest descent algorithm.[14] Then, the peptide was solvated in water within a cubic simulation box (edge = 7.8 nm) and equilibrated for 30 ns at room temperature and ambient pressure in the NPT ensemble: to this end, we have employed the Bussi-Donadio-Parrinello thermostat[15] and the Berendsen barostat,[16] with coupling constants of 0.5 and 4 ps, respectively. Periodic boundary conditions (PBCs) were applied in three dimensions and the integration time-step for the leap-frog algorithm was set to 2 fs. In parallel, a separate ice/water box was created, starting by preparing an ice crystal (of dimensions 4.7 x 5.9 x 3.9 nm) cleaved so as to have its primary prismatic face lying in the xy-plane of the simulation box: this crystal acted as the seed for the ice phase to grow along the ± z direction. The oxygen atoms within the ice slab were subjected to position restraints via a harmonic potential characterised by a spring constant of 10,000 kJ/mol; the system was then solvated, adding two layers (each one 8 nm thick) of water below and above - a total of 70818 atoms. The simulation box was elongated along the z direction to include 22.5 nm of vacuum: at this point we switched to 2D (xy) PBCs in conjunction with 9-3 Lennard-Jones "walls"[17] positioned at the top and bottom of the box. This strategy enabled us to use the Yeh and Berkowitz correction term[18] to the Ewald summation: in turn, this approach mitigates any potential artefacts due to the treatment of electrostatic

interactions when dealing with a slab geometry.[19] The cutoff for the van der Waals and electrostatic interactions was set to 12 Å and 10 Å, respectively: a switching function was used to bring the van der Waals interaction to zero at 12 Å. The geometry of the water molecules was constrained using the SETTLE algorithm[20] while the P-LINCS algorithm[21] was used to constrain the hydrogen bonds for the peptide at its equilibrium value. The ice/water system was subsequently equilibrated (NPT ensemble) at 300 K for 50 ns. Once the peptide and the ice/water system were independently equilibrated, two peptides were then placed into the ice/water box within the two water slabs, one above and one below. The system was then further equilibrated for 20 ns at 300 K and then cooled down to 265 K within 10 ns. A 200 ns production run at 265 K followed, switching to the Parrinello-Rahman barostat[22] with a coupling constant of 4 ps. Configurations were collected every 2000 MD steps.

To determine whether a certain water molecule belongs to the ice crystal or the supercooled liquid phase, we have employed a strategy based on the Steinhardt order parameters.[23] First, we compute the 6-th order Steinhardt vectors q6,m(i) as:

$$q_{6,m}(i) = \frac{\sum_{j\neq i}^{N} \sigma(|\mathbf{r}_{ij}|) Y_{6,m}(\mathbf{r}_{ij})}{\sum_{j\neq i}^{N} \sigma(|\mathbf{r}_{ij}|)}, \qquad (1)$$

where rij is the distance vector between the ith and j^{th} atom, $Y_{6,m}$ is a spherical harmonic of order {6, m} and σ is a switching function which determines the extent of the of coordination shell. Then, we combine these Steinhardt vectors to obtain the following order parameter:

$$s_6(i) = \frac{\sum_{j\neq i}^{N} \sigma(|\mathbf{r}_{ij}|) \sum_{m=-6}^{6} q_{6,m}^{*}(i) \cdot q_{6,m}(j)}{\sum_{j\neq i}^{N} \sigma(|\mathbf{r}_{ij}|)} \qquad (2)$$

where the asterisk denotes complex conjugation. By means of the clustering algorithm described in Ref.,[23] we identify the largest connected cluster of water molecules which oxygen atoms display a value of s_6(i) greater than a certain threshold (0.45, see Ref.[23] for further details). Thus, the result of this procedure provides the number of water molecules found within the largest ice cluster per trajectory frame, which invariably will be the seeded ice crystal that grows over time. Concerning the analysis of hydrogen bonds,

we have used a geometric criterion based on an acceptor-donor bond distance of less than 0.3 nm and a donor-hydrogen-acceptor angle between 160 °and 200 °. The analysis on the solvation shell around the methylene groups was based on the first minimum of the radial distribution function (g(r)) where a radial distance of 0.45 nm corresponds to the extent of the first solvation shell. Using the s_6 parameter described above we were able to distinguish which water molecules surrounding the methylene groups can be classified as either ice-like or liquid-like.

F_4 order-parameter and clathrate analysis. The average F_4 order parameter measures the H-O-O-H torsion angle between neighbouring water molecules and is defined as:

$$F_4 = \frac{1}{n}\sum_{j=1}^{N}\cos(3\varphi) \qquad (3)$$

where φ is the H-O-O-H torsion angle choosing the outermost hydrogen atoms from each molecule. F_4 values of liquid, ice and clathrate water are ~0, ~ -0.3, and ~0.7 respectively. F_4 was initially developed for sI (a well characterised clathrate structure as found in crystalline gas hydrates) but has since found useful applications for other clathrate structures too, including water[24–26]. In order to determine the water structure around the THR residues we examined the F_4 values for different sets of trajectories, i.e. trajectories where the peptide did not interact with the growing ice front, and was therefore in supercooled liquid, and trajectories where the peptide bound to ice. Additionally, different radii centred around the THR residues were also explored. The F_4 values were then computed and presented as a probability density distribution as shown in Figure S36.

Ice-tag mCherry cloning, transformation, and overexpression. The following DNA sequence was codon optimized for expression in *E. coli*, synthesized and cloned into p19AC overexpression vector by GeneArt® (ThermoFisher Scientific):
DNA sequence:
CATATGGTGAGCAAAGGTGAAGAGGATAATATGGCCATCATCAAAGAATTTATGCGCTTCAA
AGTTCACATGGAAGGTAGCGTTAATGGCCACGAATTTGAAATTGAAGGTGAAGGCGAAGGTC
GTCCGTATGAAGGCACCCAGACCGCAAAACTGAAAGTTACCAAAGGTGGTCCGCTGCCGTTTG

CATGGGATATTCTGAGTCCGCAGTTTATGTATGGTAGTAAAGCCTATGTTAAACACCCTGCAG
ATATCCCGGATTATCTGAAACTGAGCTTTCCGGAAGGTTTTAAATGGGAACGTGTGATGAAT
TTTGAAGATGGTGGTGTTGTTACCGTTACACAGGATAGCAGCCTGCAGGATGGTGAATTTAT
CTATAAAGTTAAACTGCGTGGCACGAATTTTCCGAGTGATGGTCCGGTTATGCAGAAAAAAA
CCATGGGTTGGGAAGCAAGCAGCGAACGTATGTATCCGGAAGATGGCGCACTGAAAGGTGAA
ATTAAACAGCGTCTGAAGCTGAAAGATGGCGGTCATTATGATGCAGAAGTTAAAACCACCTA
CAAAGCCAAAAAACCGGTTCAGCTGCCTGGTGCATATAACGTTAACATTAAACTGGATATCAC
CAGCCACAACGAGGATTATACCATTGTTGAACAGTATGAACGTGCAGAAGGTCGCCATAGTA
CCGGTGGTATGGATGAACTGTATAAAAGCGGTGGTGGTAAATGCTGTACCAAAAATTGTGAT
AGCACCGCACATTGCACCTAACTCGAG

Protein sequence:

MVSKGEEDNMAIIKEFMRFKVHMEGSVNGHEFEIEGEGEGRPYEGTQTAKLKVTKGGPLPFA
WDILSPQFMYGSKAYVKHPADIPDYLKLSFPEGFKWERVMNFEDGGVVTVTQDSSLQDGEFIY
KVKLRGTNFPSDGPVMQKKTMGWEASSERMYPEDGALKGEIKQRLKLKDGGHYDAEVKTTY
KAKKPVQLPGAYNVNIKLDITSHNEDYTIVEQYERAEGRHSTGGMDELYKSGGGKCCTKNCDS
TAHCT

The plasmid was transformed into *E.coli* BL21(DE3*) cells and plated onto LB agar plates with ampicillin. A single colony was picked from the plate and used to inoculate 20 mL of LB + ampicillin, which was subsequently grown overnight at 37 °C with shaking at 220 rpm. The following morning, the 20 mL overnight culture was sub-cultured in 500 mL of LB + ampicillin and grown at 37 °C with shaking at 220 rpm to an OD = 0.6. The culture was transferred to room temperature, with shaking at 220 rpm, and induced with IPTG (0.1 mM final concentration). The overexpression was continued overnight. Then, the bacteria cells were pelleted by centrifugation, showing a dark cherry pellet. The supernatant was discarded, and the pellet was resuspended in 15 mL of 1X PBS + PMSF. The cells we lysed by probe ultrasonication. The cell debris was pelleted by centrifugation and discarded. The cell lysate containing mCherry-ice tag was purified by rotary ice-affinity purification.

Rotary ice-affinity purification of mCherry-ice tag. Approximately, 15 mL of *E.coli* cell lysate containing overexpressed mCherry-ice tag was diluted to 50 mL with distilled water and cooled to 4 ^{o}C. An ice shell was made following the procedure described above. After temperature equilibration, the mCherry-ice tag cell lysate was added to the ice shell

and rotated in the ethylene glycol bath for 15 minutes. At this time, the remaining liquid was decanted and kept as the liquid fraction. The ice shell was washed with 50 mL of pre-cool distilled water to remove non-specific interactions. The ice shell was imaged under UV light and melted. The resulting liquid was termed the ice-fraction.

MALDI-TOF. MALDI-TOF experiments were performed on a 4800 MDS ScieX at the EPFL-PCL core facility. Samples were desalted and concentrated using C8/C4 zip tips and analyzed in linear positive mode using a sinapinic acid matrix.

References

1. Kong, X.-D. *et al.* De novo development of proteolytically resistant therapeutic peptides for oral administration. *Nat. Biomed. Eng.* **4**, 560–571 (2020).
2. Kather, I., Bippes, C. A. & Schmid, F. X. A Stable Disulfide-free Gene-3-protein of Phage fd Generated by In vitro Evolution. J. Mol. Biol. 354, 666–678 (2005).
3. Marshall, C. J., Basu, K. & Davies, P. L. Ice-shell purification of ice-binding proteins. *Cryobiology* **72**, 258–63 (2016).
4. Rentero Rebollo, I. & Heinis, C. Phage selection of bicyclic peptides. *Methods San Diego Calif* **60**, 46–54 (2013).
5. Congdon, T., Notman, R. & Gibson, M. I. Antifreeze (Glyco)protein mimetic behavior of poly(vinyl alcohol): Detailed structure ice recrystallization inhibition activity study. *Biomacromolecules* **14**, 1578–1586 (2013).
6. Stevens, C. A., Drori, R., Zalis, S., Braslavsky, I. & Davies, P. L. Dendrimer-Linked Antifreeze Proteins Have Superior Activity and Thermal Recovery. *Bioconjug Chem* **26**, 1908–15 (2015).
7. Guvench, O. *et al.* CHARMM Additive All-Atom Force Field for Carbohydrate Derivatives and Its Utility in Polysaccharide and Carbohydrate–Protein Modeling. *J. Chem. Theory Comput.* **7**, 3162–3180 (2011).

8. Abascal, J. L. F., Sanz, E., García Fernández, R. & Vega, C. A potential model for the study of ices and amorphous water: TIP4P/Ice. *J. Chem. Phys.* **122**, 234511 (2005).
9. Abraham, M. J. *et al.* GROMACS: High performance molecular simulations through multi-level parallelism from laptops to supercomputers. *SoftwareX* **1–2**, 19–25 (2015).
10. Klauda, J. B. *et al.* Update of the CHARMM All-Atom Additive Force Field for Lipids: Validation on Six Lipid Types. *J. Phys. Chem. B* **114**, 7830–7843 (2010).
11. Lee, H. Structures, dynamics, and hydrogen-bond interactions of antifreeze proteins in TIP4P/Ice water and their dependence on force fields. *PLOS ONE* **13**, e0198887 (2018).
12. Mochizuki, K. & Molinero, V. Antifreeze Glycoproteins Bind Reversibly to Ice via Hydrophobic Groups. *J. Am. Chem. Soc.* **140**, 4803–4811 (2018).
13. Sosso, G. C. *et al.* Unravelling the origins of ice nucleation on organic crystals. *Chem. Sci.* **9**, 8077–8088 (2018).
14. Wardi, Y. A stochastic steepest-descent algorithm. *J. Optim. Theory Appl.* **59**, 307–323 (1988).
15. Bussi, G., Donadio, D. & Parrinello, M. Canonical sampling through velocity rescaling. *J. Chem. Phys.* **126**, 014101 (2007).
16. Berendsen, H. J. C., Postma, J. P. M., van Gunsteren, W. F., DiNola, A. & Haak, J. R. Molecular dynamics with coupling to an external bath. *J. Chem. Phys.* **81**, 3684–3690 (1984).
17. Abraham, F. F. & Singh, Y. The structure of a hard-sphere fluid in contact with a soft repulsive wall. *J. Chem. Phys.* **67**, 2384–2385 (1977).
18. Yeh, I.-C. & Berkowitz, M. L. Ewald summation for systems with slab geometry. *J. Chem. Phys.* **111**, 3155–3162 (1999).
19. Bostick, D. & Berkowitz, M. L. The implementation of slab geometry for membrane-channel molecular dynamics simulations. *Biophys. J.* **85**, 97–107 (2003).

20. Miyamoto, S. & Kollman, P. A. Settle: An analytical version of the SHAKE and RATTLE algorithm for rigid water models. *J. Comput. Chem.* **13**, 952–962 (1992).

21. Hess, B. P-LINCS: A Parallel Linear Constraint Solver for Molecular Simulation. *J. Chem. Theory Comput.* **4**, 116–122 (2008).

22. Parrinello, M. & Rahman, A. Polymorphic transitions in single crystals: A new molecular dynamics method. *J. Appl. Phys.* **52**, 7182–7190 (1981).

23. Tribello, G. A., Giberti, F., Sosso, G. C., Salvalaglio, M. & Parrinello, M. Analyzing and Driving Cluster Formation in Atomistic Simulations. *J. Chem. Theory Comput.* **13**, 1317–1327 (2017).

24. Parui, S. & Jana, B. Factors Promoting the Formation of Clathrate-Like Ordering of Water in Biomolecular Structure at Ambient Temperature and Pressure. *J. Phys. Chem. B* **123**, 811–824 (2019).

25. Rodger, P. M., Forester, T. R. & Smith, W. Simulations of the methane hydrate/methane gas interface near hydrate forming conditions conditions. *Fluid Phase Equilibria* **116**, 326–332 (1996).

26. Nguyen, A. H., Koc, M. A., Shepherd, T. D. & Molinero, V. Structure of the Ice–Clathrate Interface. *J. Phys. Chem. C* **119**, 4104–4117 (2015).

27. Hafsa, N. E., Arndt, D. & Wishart, D. S. CSI 3.0: a web server for identifying secondary and super-secondary structure in proteins using NMR chemical shifts. *Nucleic Acids Res.* **43**, W370–W377 (2015).
